# TNF receptor-associated factors: promising targets of natural products for the treatment of osteoporosis

**DOI:** 10.3389/fphys.2025.1527814

**Published:** 2025-05-27

**Authors:** Xicheng Yang, LiLi Zhao, YinQuan Pang

**Affiliations:** ^1^ Graduate School, Hebei Medical University, Shijiazhuang, Hebei, China; ^2^ Orthopedics and Arthrology, People Hospital of Xingtai, Xingtai, Hebei, China; ^3^ Graduate School, Chengde Medical University, Chengde, Hebei, China

**Keywords:** common genes, such as SNAP25, AQP4, SV2B, KCND3, ABCA2, CD163, CD14

## Abstract

Tumor necrosis factor receptor-associated factors (TRAFs) are crucial intracellular signaling proteins in bone homeostasis. TRAFs mediate pathways associated with bone remodeling, particularly in response to inflammatory stimuli, influencing osteoclast differentiation and function. Dysregulation of TRAF-mediated signaling contributes significantly to osteoporosis, a condition marked by increased bone resorption and fragility. Natural products, with their anti-inflammatory and antioxidant properties, offer promising therapeutic potential by targeting TRAF-associated pathways to inhibit excessive osteoclast activity and promote bone formation. This review explores the mechanisms by which natural compounds modulate TRAF signaling in osteoclastogenesis and osteoblastogenesis, providing insights into their potential for osteoporosis treatment.

## Graphical Abstract

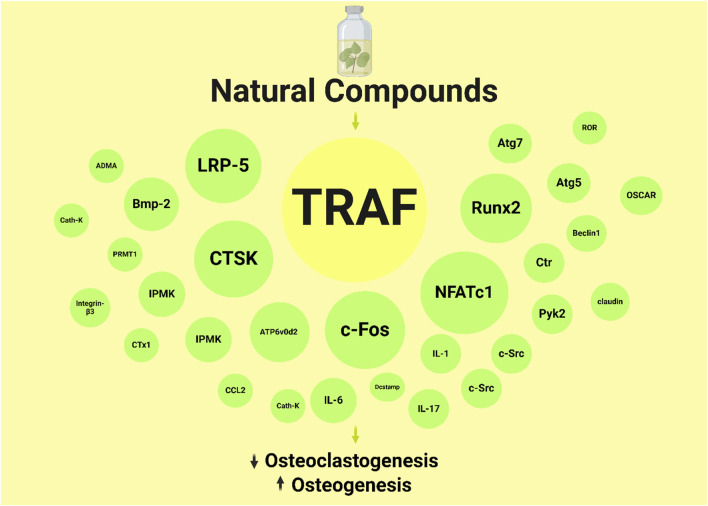

## 1 Introduction

Osteoporosis is a chronic, progressive bone disorder marked by attenuated bone mineral density (BMD) and structural deterioration of bone tissue, posing an elevated risk for fractures, predominantly in the elderly population. The primary pathophysiological mechanism of osteoporosis is an imbalance between bone resorption and bone formation, resulting in net bone loss (Salari et al., 2021). The activities of osteoclasts, the bone-resorbing cells, and osteoblasts, the bone-forming cells, primarily drive this imbalance. Under physiological conditions, bone remodeling maintains skeletal integrity through a well-coordinated cycle involving the differentiation, activation, and apoptosis of osteoclasts and osteoblasts. However, dysregulated signaling cascades in osteoporosis lead to excessive osteoclast activity and impaired osteoblast function, weakening bones (Zhang et al., 2024a).

Among the molecular regulators involved in bone remodeling, tumor necrosis factor receptor-associated factors (TRAFs) have emerged as key players. TRAFs are adapter proteins that mediate intracellular signaling cascades from various receptors, including those in the TNF receptor superfamily (Kitaura et al., 2022). TRAFs, especially TRAF2, TRAF3, TRAF5, and TRAF6, are integral to bone metabolism as they modulate vital pathways such as nuclear factor-kappa B (NF-κB), mitogen-activated protein kinases (MAPKs), and phosphoinositide 3-kinase/protein kinase B (PI3K/AKT). These pathways are crucial in regulating osteoclastogenesis (the formation of osteoclasts) and osteoblastogenesis (the induction of osteoblastic differentiation), and their dysregulation is associated with the pathogenesis of osteoporosis (Li et al., 2017; Xiao et al., 2023; Shi and Sun, 2018). TRAF6, in particular, acts as a major factor in the receptor activator of the nuclear factor kappa-B ligand (RANKL) signaling pathway, a critical pathway for osteoclast differentiation and function. Elevated RANKL levels, as observed in osteoporosis, lead to enhanced TRAF6-mediated signaling, promoting osteoclastogenesis and bone resorption (Yu et al., 2021; Li et al., 2024; Chen et al., 2019).

Current investigations have highlighted the potential value of natural products in modulating TRAF-mediated pathways as a therapeutic strategy for osteoporosis. Natural compounds—often with antioxidant, anti-inflammatory, and bone-protective properties—target these signaling pathways to impede osteoclast activity or promote osteoblast capability, thereby restoring bone balance (Şöhretoğlu and Renda, 2020). For example, polyphenols such as resveratrol and curcumin have been revealed to repress TRAF-mediated NF-κB and MAPK signaling, reducing osteoclast differentiation and activity. Similarly, other bioactive compounds, including flavonoids and terpenoids, interact with TRAF pathways to regulate oxidative stress, inflammation, and bone cell metabolism (Du et al., 2024; Xue et al., 2023; Ke et al., 2020).

Given the complexity of osteoporosis and the limitations of conventional treatments, natural products offer a favorable, multifaceted approach to managing this disease. This review discusses the role of TRAFs in bone metabolism and the potential of natural compounds to target TRAF-associated pathways. By examining the molecular mechanisms by which natural products influence TRAF signaling, we aim to highlight novel therapeutic strategies for osteoporosis that leverage the bioactivity of plant-derived compounds, providing a foundation for future clinical applications.

## 2 Bone remodeling dynamics

**FIGURE 1:**
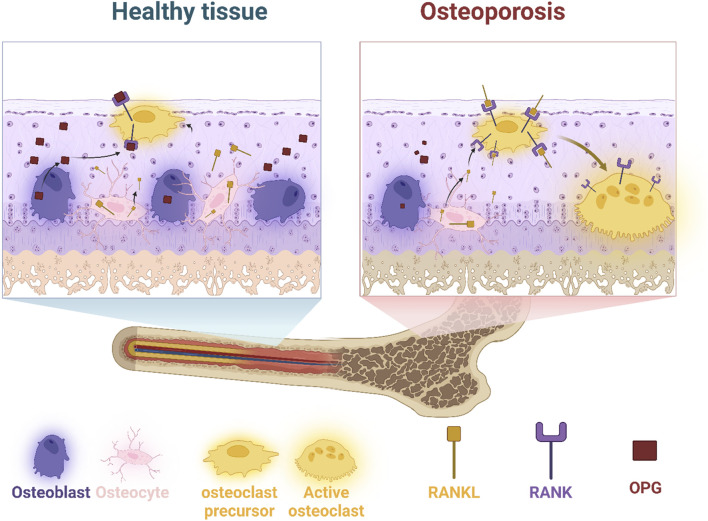
Comparison of healthy and osteoporotic bone tissue highlights the cellular interactions and signaling pathways involved in bone remodeling. Osteoblasts (bone-forming cells), osteocytes (mature bone cells), and osteoclast precursors are in balance in healthy tissue. However, an increase in RANKL in osteoporosis promotes osteoclast activation, leading to excessive bone resorption. Osteoprotegerin (OPG) acts as a decoy receptor to regulate RANKL activity, but its diminished function in osteoporosis results in bone deterioration.

Bone remodeling is a multifaceted procedure orchestrated by a myriad of cellular and molecular mechanisms. It encompasses five distinct phases that seamlessly transition from one to the next to maintain bone homeostasis. The activation phase initiates bone remodeling in response to local mechanical or hormonal signals, with osteocytes serving as the sentinel cells that detect and translate these cues into biological reactions within the bone matrix. This phase is characterized by the orchestration of various local and systemic regulators, including transforming growth factor-beta (TGF-β), macrophage colony-stimulating factor (M-CSF), and RANKL. These factors collectively stimulate osteoclastogenesis and kick-start a fresh cycle of bone remodeling (Chen Q. et al., 2022; Kim H-J. et al., 2022; Šromová et al., 2023). Following activation, in the resorption phase, mature osteoclasts actively digest the bone matrix’s mineral and organic components. This process involves the secretion of matrix metalloproteinases (MMPs), which carve out resorption lacunae beneath the canopy cells. As resorption progresses, the bone microenvironment undergoes dynamic changes that pave the way for subsequent phases (Figure 1) (Zhu et al., 2020; Bagheri et al., 2023).

The reversal phase marks a transition as mature osteoclasts undergo apoptosis, making way for osteoblasts to migrate to the resorption site. Local molecules such as TGF-β serve as a cornerstone in this phase by attracting osteoblasts and initiating bone formation (Kawai et al., 2023). This sets the stage for the formation phase, where osteoblasts take center-stage in orchestrating bone remodeling. Over the course of several months, osteoblasts deposit organic bone matrix, primarily composed of type I collagen, to restore bone integrity (Delaisse, 2014; Schlesinger et al., 2019). During the termination phase, an equilibrium is established when bone formation and resorption proceed at equivalent rates. This phase is characterized by the cessation of bone formation activity, with osteoblasts either undergoing apoptosis or differentiating into new osteocytes. Simultaneously, bone mineralization commences, completing the remodeling process (Maciel et al., 2023). Key cellular players in bone remodeling include osteoclasts, osteoblasts, and osteocytes. Osteoclasts, differentiated from hematopoietic stem cells, undergo a series of differentiation and maturation steps orchestrated by signaling pathways such as M-CSF/c-fms and RANKL/RANK/OPG (Di et al., 2024). On the other hand, osteoblast differentiation from mesenchymal stem cells is governed by transcription factors like Runx2, osterix, and β-catenin, with the Wnt pathway playing a central role. Osteocytes, as the most prevalent bone cells, act as master regulators of bone remodeling by secreting cytokines and sensing mechanical stimuli (Adejuyigbe et al., 2023).

Understanding the intricacies of these cellular and molecular mechanisms is crucial for unraveling the pathophysiology of bone disorders like osteoporosis and developing targeted therapeutic interventions to mitigate their impact.

## 3 Pathophysiology of osteoporosis

Osteoporosis is a complex skeletal disorder indicative of reduced BMD and deterioration of bone microarchitecture, causing enhanced bone fragility and susceptibility to fractures. The pathophysiology of osteoporosis involves a disruption in the delicate balance between osteoclastic bone resorption and osteoblastic bone formation within the process of bone remodeling.

### 3.1 Hormonal factors

Hormonal regulation is crucial for sustaining bone homeostasis, with estrogen being particularly significant in preserving bone density. It achieves this by suppressing osteoclast activity and enhancing osteoblast function. A deficiency in estrogen levels post-menopause is a dominant factor for osteoporosis development in women. Although the decline in bone density due to estrogen deficiency influences both men and women, it is especially severe in postmenopausal women because of the sharp decrease in estrogen levels.

The role of estrogen in maintaining bone homeostasis primarily involves its interaction with estrogen receptors estrogen receptor-α (ERα) and ERβ, which are present within diverse cell types, including osteocytes, osteoblasts, bone marrow-derived mesenchymal stem cells (BM-MSCs), and osteoclasts (Kondoh et al., 2014; Soltanyzadeh et al., 2020). This interaction activates the Wingless integrated type-1 (Wnt)/β-catenin signaling pathway, promoting the proliferation and differentiation of osteoblasts. Research shows that decreasing the expression of ERα hampers osteoblast differentiation, underscoring the criticality essentially of ERα in bone regeneration and fracture healing (Shi and Morgan, 2024).

Additionally, estrogen inhibits the secretion of a range of inflammatory cytokines, including tumor necrosis factor-α (TNF-α); interleukins (IL)-1, -4, -6; and interferon-γ (IFN-γ), which participate in osteoclast development and bone resorption. Estrogen also reduces the secretion of the receptor activator of nuclear factor kappa-Β ligand (RANKL), while promoting the release of factors that inhibit osteoclast activity, comprising growth hormone, glucagon-like peptide-1 (GLP-1), and osteoprotegerin (OPG), thereby limiting osteoclast function. During estrogen deficiency, the activation of IL-17 significantly contributes to osteoporosis by increasing the secretion of RANKL, TNF-α, and IL-1 and IL-6, promoting bone resorption. Moreover, sustained generation of pro-inflammatory cytokines TNF-α and IL-17 due to estrogen deficiency enhances RANKL-induced osteoclastogenesis by transforming memory T-cells into effector cells. Estrogen deficiency also affects T-cell immune factors, leading to increased TNF-α production by T cells, further stimulating osteoclastic bone resorption and bone loss (Zha et al., 2018; Huang et al., 2024; Tai-Hung et al., 2010; Funaki et al., 2018). In conclusion, estrogen is imperative for maintaining bone equilibrium through the regulation of various immune and cellular mechanisms. Similarly, androgens are fundamental in maintaining bone health in men, and reduced levels are associated with bone loss. Parathyroid hormones also influence bone remodeling by stimulating osteoclast activity and mobilizing calcium from bone (Sun P. et al., 2020).

### 3.2 Genetic contributions to osteoporosis

Osteoporosis is a condition impacted by a combination of genetic and environmental factors. While inherited physiological traits play a significant role, it is not solely a hereditary disease. Genetic factors contribute up to 85% to the development of osteoporosis, with multiple alleles inherited from family members being a crucial risk factor. However, metabolic, dietary, and other elements, like vitamin D levels, play pivotal roles (Pouresmaeili et al., 2018). Rarely, single-gene mutations can directly impact bone health, affecting bone mineralization and the function of bone-forming cells (osteoblasts). Some mutations in genes that participate in WNT1 signaling, like WNT1, can lead to early-onset osteoporosis and osteogenesis imperfecta (Lu et al., 2018).

More commonly, osteoporosis arises from a complex genetic architecture involving multiple mutations. Genome-wide association studies (GWAS) have recognized various loci connected to BMD, with certain genes like COL1A1 and COL1A2, which encode type I collagen, being particularly significant. Other genes, such as CLCN7, GALNT3, IBSP, LTBP3, RSPO3, SOST, and SOX4, also influence BMD (Koller et al., 2010; Trajanoska and Rivadeneira, 2019; Zeng et al., 2024). Several genes identified through GWAS, including TNFRSF11B, LRP5, RUNX2, SP7, SOST, DKKI, and ESR1, strongly impact osteoporosis development. Additionally, rare genetic variants and low-frequency mutations can contribute to decreased BMD and fracture risk (Qin et al., 2016).

**FIGURE 2:**
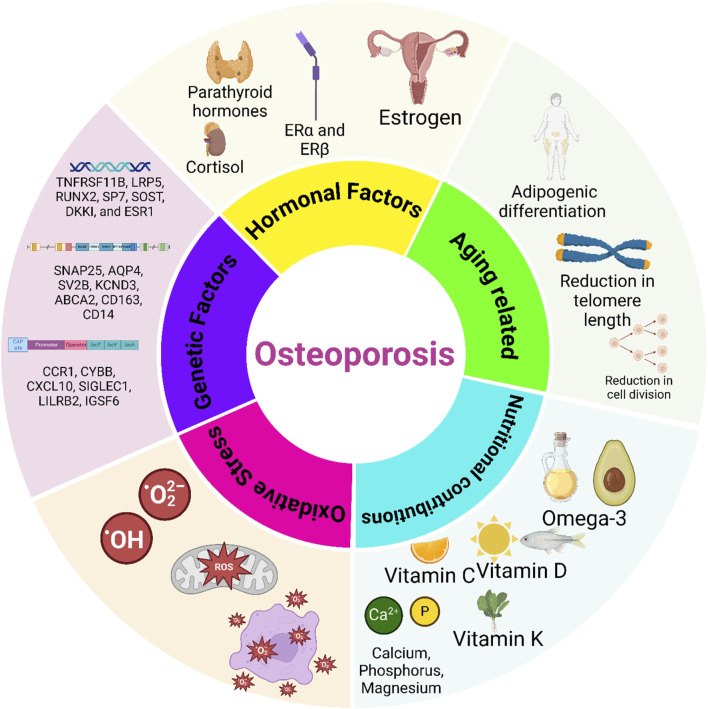
Key factors influencing osteoporosis development. Hormonal factors, such as estrogen deficiency, play a crucial role in bone loss by enhancing osteoclast activity and reducing bone density, particularly in postmenopausal women. Genetic factors, through specific gene mutations and inherited traits, contribute to susceptibility by influencing bone mineral density (BMD) and osteoblast function. Nutritional factors, including calcium, vitamin D, protein, and other micronutrients, support bone health, while deficiencies elevate osteoporosis risk. Age-related changes and oxidative stress also contribute by disrupting bone remodeling and cellular function, with oxidative stress promoting osteoclast activity and cell senescence. Understanding these interconnected mechanisms can guide targeted therapies for osteoporosis prevention and management.

Interestingly, correlations exist between osteoporosis and other conditions. Gene expression profiling databases have revealed associations between osteoporosis and Parkinson’s disease, as well as between chronic gastritis and inflammatory bowel diseases like Crohn’s disease. Common genes, such as SNAP25, AQP4, SV2B, KCND3, ABCA2, CD163, CD14, CCR1, CYBB, CXCL10, SIGLEC1, LILRB2, IGSF6, and MS4A6A, have diagnostic value across these conditions (Figure 2) (Zhivodernikov et al., 2023).

### 3.3 Nutritional factors involving osteoporosis

Sufficient nutritional intake, particularly of calcium, vitamin D, and other micronutrients, is imperative for maintaining optimal bone health. Inadequacies in nutrition can impede bone mineralization and remodeling processes, thereby exacerbating osteoporosis risk (Migliaccio et al., 2023). Calcium and vitamin D are pivotal for bone health as they constitute integral components of the bone mineral matrix, primarily as calcium phosphate. Although dietary sources are preferred for obtaining calcium, supplementation becomes necessary in cases of insufficient dietary intake or impaired absorption. Primary dietary sources of calcium include dairy products, fish, legumes, and select vegetables and fruits. More importantly, avoiding the consumption of substances impairing calcium uptake, such as nicotine, is necessary for bone mineralization (Fang et al., 2024; Torfadóttir and Uusi-Rasi, 2023). Vitamin D is chiefly synthesized through exposure to sunlight, with limited dietary sources, like oily fish, mushrooms, and dairy products with added nutrients. Adequate levels of vitamin D are particularly crucial for bone health, especially among elderly individuals or postmenopausal women, and for enhancing the effectiveness of anti-osteoporotic medications (Migliaccio et al., 2023; Polzonetti et al., 2020; Andreo-López et al., 2023). Dairy products, rich in essential nutrients including calcium, phosphorus, magnesium, protein, and, if fortified, vitamin D, confer notable benefits for bone health. Consistent consumption of dairy products has been correlated with elevated BMD and reduced fracture incidence in various research studies (Hidayat et al., 2022). Furthermore, fermented dairy products offer prebiotics and probiotics that can augment calcium absorption and bone metabolism (Harahap et al., 2024; Yumol et al., 2025). Nevertheless, research yields inconclusive findings on the benefits of high dairy intake, potentially due to variations in dietary habits, policies regarding vitamin D fortification, and demographic characteristics among study populations.

Potassium and magnesium also have a significant influence on bone health. Potassium aids in maintaining an alkaline state within the body, thereby reducing calcium loss from bones and promoting calcium retention in the kidneys. Meanwhile, magnesium is indispensable for calcium metabolism, influencing bone formation and structure. Green leafy vegetables, legumes, and nuts are prominent sources of magnesium. Magnesium deficiency has been linked to diminished bone formation and heightened bone fragility, thereby contributing to osteoporosis (Singh Poonam and Kushwaha, 2024; Fouhy et al., 2023). Protein, constituting approximately half of bone volume, is indispensable for bone integrity. Adequate dietary protein is requisite for preserving bone mass and strength as it modulates the generation and function of insulin-like growth factor I (IGF-I), which is pivotal for bone formation. Despite concerns regarding high protein intake leading to calcium loss, recent studies suggest that higher protein consumption supports superior BMD and reduced fracture risk, particularly when combined with the intake of sufficient calcium (Groenendijk et al., 2023; Zhu et al., 2011). Vitamin K is fundamental to bone matrix formation, facilitating mineralization. Despite evidence from some meta-analyses pointing to limited effects on BMD and fracture outcomes, vitamin K is still a key nutrient for bone health (Atkins et al., 2009). Additionally, vitamin C, with its antioxidant properties, supports osteoblast activity and collagen formation, thereby positively impacting BMD and reducing fracture risk (Figure 2) (Wang and Ma, 2023). The interplay of nutrients in bone health is intricate and multifaceted. While the significance of calcium and vitamin D is well-established, the contributions of other nutrients such as potassium, magnesium, protein, vitamins K and C, omega-3 polyunsaturated fatty acids (PUFAs), and zinc are also noteworthy (Skalny et al., 2024). Deficiencies in these nutrients contribute to osteoporosis, drawing attention to the importance of incorporating these essential components into a balanced diet. Recommendations regarding nutrition should be grounded in a comprehensive understanding of individual dietary patterns, lifestyle factors, and available scientific evidence.

### 3.4 Age-related changes

Aging is the primary non-modifiable risk factor for osteoporosis. With advancing age, bone remodeling becomes less efficient, leading to gradual bone loss. Age-related hormonal changes, such as decreased estrogen and testosterone levels, further exacerbate bone loss and fragility. Presently, the primary cause of cellular aging is attributed to the gradual reduction in telomere length. Alongside the natural limit to cell replication (replicative senescence), a range of detrimental impacts, notably stemming from interactions with reactive oxygen species (ROS), contribute to DNA deterioration and trigger the immune system’s response to such genetic damage (Passos et al., 2007; Ma et al., 2019; Baek et al., 2010). These factors activate pathways, such as p53/p21 Cip1 and p16 Ink4a, inhibiting cell division. With aging, the levels of the senescence biomarker p16 Ink4a and senescence-associated secretory phenotype (SASP) increase in osteocytes, which results in decreased osteogenic differentiation and a shift toward adipogenic differentiation in bone marrow stem cells (BMSCs) (Yan et al., 2024). A recent study comparing young and old mice found that older BMSCs exhibit a reduced ability to differentiate into osteoblasts, as indicated by the lower expressions of markers like RUNX2, DLX5, collagen-1, and osteocalcin, and diminished calcium accumulation. Similar age-related declines were seen in Wistar rats and human BMSCs from adipose tissue, with younger individuals showing higher osteomarker expression and mineralization (Qin et al., 2021).

Furthermore, aging leads to higher expression of adipogenic genes, such as PPAR-γ2, which shifts the balance toward adipogenesis rather than osteogenesis in BMSCs (Han et al., 2019). Use of senolytics or senomorphics has shown potential in preserving bone mass and reducing cellular senescence. For example, the senolytic drug AP20187 increased bone mass and decreased senescence markers in aged mice (Doolittle et al., 2021). These results indicate the potential of targeting cellular senescence with these treatments to help maintain bone health in aging people.

### 3.5 Oxidative stress

Oxidative stress contributes significantly to the aging process and the development of various health conditions, including neurological, cardiovascular, and metabolic diseases. It is caused when the production of reactive oxygen species (ROS) exceeds the body’s ability to counteract them with antioxidants. ROS, which are generated during cellular metabolism and ATP synthesis, accumulate excessively, causing oxidative stress and the onset of disease. ROS damages cellular and mitochondrial membranes, triggering programmed cell death pathways (Jomova et al., 2023; Mao et al., 2018). The aging process exacerbates oxidative stress by impairing mitochondrial function, producing increased ROS. Mitochondrial DNA (mtDNA) is especially susceptible to damage from oxidative stress due to its limited protective mechanisms compared to nuclear DNA. Research has shown that cellular senescence, oxidative stress, and mitochondrial dysfunction are interconnected, with oxidative stress playing a pivotal role in disease progression (Lee et al., 2023; Martínez-Cisuelo et al., 2016).

In the context of osteoporosis, oxidative stress negatively affects bone formation by fostering the death of osteocytes and osteoblasts, disrupting the normal bone remodeling process. Specifically, oxidative stress hinders osteoblast differentiation through the extracellular signal-regulated kinase (ERK)-NF-κB signaling axis (Hsieh et al., 2010). Cellular defense mechanisms, such as glutathione peroxidase and transforming growth factor β (TGF-β), attempt to protect osteoblasts from ROS-induced damage. Moreover, oxidative stress enhances the differentiation and activity of osteoclasts, leading to intensified bone resorption. The balance of reduced glutathione (GSH) to oxidized glutathione (GSSG) is vital for regulating osteogenic differentiation and bone formation (Xia et al., 2024). Clinical studies have demonstrated a link between elevated ROS levels, advanced glycation end products (AGEs), and age-related musculoskeletal disorders, including reduced skeletal mineralization and muscle strength (Zhang X. et al., 2024). In therapeutic approaches, antioxidants, particularly melatonin, promise to mitigate osteoporosis. Melatonin scavenges ROS, increases the synthesis of antioxidant enzymes, and promotes osteogenic differentiation. Additionally, melatonin suppresses osteoclast formation and enhances the expression of sirtuin 1 (SIRT1), leading to improved bone mass in preclinical models (Wu et al., 2024; Chen W. et al., 2020). Research into oxidative stress-related mechanisms in osteogenesis suggests potential therapeutic interventions using antioxidants, including phytochemicals (Kim E-N. et al., 2022). Mechanistic studies, such as ferroptosis-mediated cell death of osteocytes and osteoblasts due to iron-induced lipid peroxidation, contribute to our understanding of oxidative stress in osteoporosis (Jiang et al., 2022). Vitamin K2 has emerged as a protective factor against osteocyte death through the AMP-activated protein kinase (AMPK)/SIRT1 signaling pathway, highlighting the crucial role of oxidative stress in osteoporosis pathology (Liu P. et al., 2022).

In summary, osteoporosis results from a complex interplay of factors involving dysregulated bone remodeling, hormonal imbalances, genetic predisposition, nutritional deficiencies, lifestyle factors, and age-related changes. Gaining insights into these mechanisms is important for developing targeted interventions aimed at preventing and managing osteoporosis and reducing the associated morbidity and mortality.

## 4 Structure of TRAFs

In the context of the TRAF family of proteins, an essential component of intracellular signaling pathways, the defining feature is the TRAF domain, a protein-interacting domain comprising approximately 180 amino acids. Among mammalian species, six out of the seven known TRAF proteins (TRAF1–TRAF6) possess this characteristic (Jang et al., 2024). Structurally, the TRAF domain is known to comprise two distinct regions: the TRAF-N and TRAF-C domains. Although the TRAF-C domain predominantly interacts with various receptors, the TRAF-N domain is a binding site for many intracellular signaling molecules. Despite the overall structural similarity among TRAF domains, each member of the TRAF family exhibits specific biological functions dictated by the repertoire of interacting partners, including upstream receptors and downstream effectors. Detailed structural investigations have revealed nuanced differences among TRAF family members. Notably, variations in the length and positioning of specific loops, particularly those connecting β5–β6 and β6–β7, distinguish TRAF4 and TRAF6 from their counterparts. Moreover, the localization of the TRAF-N coiled-coil domain differs across TRAF family members, with TRAF4 exhibiting an outer layer positioning, suggesting potential functional implications (Park, 2018; Park, 2021).

Electrostatic surface analysis further delineates distinctions within the TRAF family. Although TRAF1, TRAF2, TRAF3, and TRAF5 share similar electrostatic features, enabling accommodation of diverse receptors within a common binding pocket, TRAF4 and TRAF6 exhibit unique surface characteristics, indicative of their ability to engage with receptors through distinct modes of interaction (Park, 2021). Functionally, the TRAF domain forms a stable trimeric structure in solution, a configuration crucial for its cellular activities. This trimeric assembly, characterized by a mushroom-like shape, with the TRAF-C domain forming the cap and the TRAF-N coiled-coil domain forming the stalk, facilitates interactions with various cellular components. Key interaction sites within the TRAF domain, particularly involving β3, β4, β6, and β7, are implicated in receptor engagement. Theoretical models integrating the trimeric TRAF domain with the zinc-finger domain and RING domain have provided insights into the organization of full-length TRAF structures. Notably, while the C-terminal TRAF domain forms a functional trimer engaging with active receptors, the N-terminal RING domain and zinc-finger domain retain flexibility, potentially contributing to the adaptability of TRAF-mediated signaling pathways (Xie, 2013; So, 2022).

In summary, the structural and functional analyses underscore the complexity and versatility of TRAF proteins in mediating intracellular signaling events, with implications for understanding various physiological and pathological processes.

## 5 TRAF mechanism of action in osteoporosis

### 5.1 TRAFs and NF-κB signaling: a central regulator of osteoclast differentiation

One of the key pathways in which TRAFs are involved is the nuclear factor kappa-light-chain-enhancer of activated B cells (NF-κB) pathway. The interaction between TRAFs and NF-κB is critical in a range of physiological and disease-related processes, including osteoporosis. Osteoporosis results from an imbalance between bone resorption and bone formation. Osteoclasts, the cells responsible for bone resorption, and osteoblasts, responsible for bone formation, must maintain a delicate balance (Abu-Amer, 2013). The NF-κB pathway is imperative in this regulation, and TRAFs are critical modulators of this pathway. The NF-κB pathway is triggered by various stimuli, including inflammatory cytokines like TNF-α and IL-1. TRAFs are recruited to the receptor complexes when these cytokines bind to their receptors. For instance, TRAF2 and TRAF6 are prominently involved in TNFR1 and IL-1 receptor signaling pathways. These TRAFs act as scaffold proteins, enabling the assembly of signaling complexes that activate downstream kinases like IκB kinase (IKK). IKK activation results in the phosphorylation and degradation of IκB proteins, which generally inhibit NF-κB signaling by sequestering it in the cytoplasm. The degradation of IκB allows NF-κB dimers to relocate to the nucleus, where they activate the transcription of various genes involved in inflammation, immune responses, and cell survival. In bone metabolism, NF-κB activation promotes the expression of genes such as RANKL and macrophage colony-stimulating factor (M-CSF), necessary for osteoclast differentiation and function (Han et al., 2003; Zheng et al., 2024; Hu et al., 2017; Xu et al., 2020).

Following menopause, estrogen deficiency can increase levels of pro-inflammatory cytokines, M-CSF, and RANKL. This elevation subsequently enhances the maturation of osteoclasts originating from bone marrow monocytes (BMMs) (Almeida et al., 2017). TRAF6 plays a particularly indispensable role in RANKL-mediated osteoclastogenesis. When RANKL binds to its receptor RANK on osteoclast precursors, TRAF6 is recruited to the receptor complex. This recruitment is necessary for activating NF-κB signaling and its downstream signaling pathways, comprising NFATc1, TRAP, CTR, MMP-9, and cathepsin K. The activation of these pathways leads to the differentiation of osteoclast precursors into mature osteoclasts, which are accountable for bone resorption (Zhai et al., 2020). Moreover, TRAF6 can interact with other signaling molecules and pathways that impact bone metabolism. Recent research has established the integral role of annexin A3 (ANXA3) in osteoclastogenesis. ANXA3 is a member of the annexin family, predominantly expressed in myeloid cell lineages. This protein, comprising four conserved domains, is vital for membrane transport and calmodulin-dependent functions and contributes to various pathological and physiological conditions (Gerke et al., 2024; Yang et al., 2021). ANXA3 directly interacts with RANK, enhancing its transcription and preventing its degradation via the ubiquitin-proteasome pathway, stabilizing the RANK protein. Additionally, ANXA3 binds directly to TRAF6, promoting its transcription and abrogating its degradation through both the autophagy-lysosomal and ubiquitin-proteasome pathways, stabilizing the TRAF6 protein. ANXA3-mediated upregulation of TRAF6 facilitates the phosphorylation of NF-κB subunit P65, which in turn augments the expression of osteoclast-specific genes in osteoclast precursors, including Acp5, Ctsk, and Mmp9 (Lin et al., 2023). The anti-inflammatory protein A20, also referred to as tumor necrosis factor alpha-induced protein 3 (TNFAIP3), is instrumental in regulating NF-κB signaling and the expression of inflammatory genes. In mice, myeloid-specific deficiency of A20 leads to the unexpected emergence of severe destructive polyarthritis, exhibiting many characteristics of rheumatoid arthritis. Recent investigations have revealed that A20 is also implicated in various osteoporotic phenotypes (Ma and Malynn, 2012; Momtazi et al., 2019). Within minutes of ligand binding, A20 is recruited to the RANK receptor complex, inhibiting NF-κB activation. This inhibition occurs independently of A20’s deubiquitinating activity and is mediated through its ubiquitin-binding zinc finger domains 4 and 7 and abrogates the transcription of osteoclast-specific genes such as TRAP, Ctsk, NFATc1, cFms, and OPG (Martens et al., 2022). Moreover, despite the distinct function of TRAF6 in the RANKL signaling, TRAF family member-associated NF-κB activator (TANK) itself has drawn much attention due to its substantial negative role in RANKL-related bone homeostasis and osteoclastogenesis. TANK performed its function by binding with all known TRAF proteins, serving as a modulator of TRAF-related signaling (Maruyama et al., 2012). In macrophages and B cells from TANK-deficient (Tank−/−) mice, there is an enhanced activation of NF-κB and AP-1 following stimulation of B-cell receptor (BCR) and TLRs. This is accompanied by increased TLR- and BCR-related ubiquitination of TRAF6 in Tank−/− macrophages, indicating that TANK plays a role in inhibiting TRAF6 activation downstream of TLRs (Kawagoe et al., 2009). Researchers have attained a more profound comprehension of this phenomenon in osteoporosis as a recent study found that during RANKL-mediated osteoclastogenesis, TANK expression was upregulated, and its overexpression resulted in diminished osteoclast formation. In addition, macrophages lacking TANK (Tank−/−) exhibited heightened osteoclastogenesis, attributed to augmented ubiquitination of TRAF6, stimulation of the canonical NF-κB pathway, as well as increased NFATc1 and c-Fos activation (Maruyama et al., 2012).

In osteoporosis, the overactivation of the NF-κB pathway contributes to excessive osteoclast activity and increased bone resorption. Elevated levels of inflammatory cytokines such as TNF-α and IL-1 in the bone microenvironment enhance the recruitment and activation of TRAFs, thereby amplifying NF-κB signaling. This heightened signaling increases the expression of osteoclastogenic factors, leading to greater bone resorption and a net loss of bone density and structural integrity. However, NF-κBs and TRAFs may serve as negative regulators during osteoporosis and reverse osteoclast formation. In this regard, Yao et al. revealed an unanticipated role of TNF in limiting both TNF- and RANKL-related osteoclastogenesis by promoting the expressions of NF-κB p100 and TRAF3 in osteoclast precursors. Furthermore, TRAF3 prevents p100 from undergoing proteasomal processing to p52 by intensifying the proteasomal degradation of NF-κB–inducing kinase (NIK) through its direct physical interaction with the TRAF3 sequence binding motif in NIK, therefore limiting bone destruction and osteoclastogenesis-mediated bone loss (Yao et al., 2009).

The comprehension of the specific roles and mechanisms of different TRAF family members in bone metabolism can provide insights into potential therapeutic targets for osteoporosis. For instance, targeting TRAF6 or modulating its interaction with RANK could potentially reduce excessive osteoclast activity and bone resorption. Similarly, enhancing TRAF3 function might help inhibit osteoclastogenesis and mitigate bone loss. In summary, TRAFs are essential regulators of the NF-κB signaling pathway, which plays a significant role in bone resorption and formation. The interaction between TRAFs and NF-κB is a crucial mechanism underlying the pathogenesis of osteoporosis. By promoting the activation of NF-κB and the expression of osteoclastogenic factors, TRAFs contribute to excessive bone resorption characteristic of osteoporosis. Therapeutic strategies targeting TRAF-mediated signaling pathways may offer new avenues for the treatment and prevention of osteoporosis.

### 5.2 MAPK pathway and TRAF-mediated osteoclastogenesis

The interaction between TRAFs and mitogen-activated protein kinases (MAPKs) plays a significant role in the pathogenesis of osteoporosis. This intricate interplay is essential for comprehending the molecular mechanisms driving bone resorption and formation balance and how disruptions in these processes lead to osteoporosis (Wang M. et al., 2022). MAPKs, including ERK, c-Jun N-terminal kinases (JNK), and p38 MAPK, are critical in transducing extracellular signals into intracellular responses. Upon activation by TRAF6, MAPKs are phosphorylated and subsequently activate various transcription factors, such as activator protein-1 (AP-1) and NF-κB, which are essential for the differentiation and activity of osteoclasts (Tan et al., 2017).

The MAPK pathway’s activation through TRAFs involves a series of phosphorylation events. TRAF6, through its ubiquitin ligase activity, mediates the ubiquitination of key signaling molecules like TAK1 (TGF-beta-activated kinase 1). This modification allows TAK1 to phosphorylate and activate the MAPK kinase kinases (MAP3Ks), which in turn phosphorylate and stimulate MAPK kinases (MAP2Ks). Finally, MAP2Ks phosphorylate MAPKs, leading to their activation (Chen Y. et al., 2022). The binding of RANKL to the RANK receptor on the cell surface leads to the formation of RANKL/RANK/TRAF complexes, which subsequently trigger the MAPK pathway. Kim and colleagues carried out Western blot analysis on RAW 264.7 cells and revealed that the stimulated RANKL/RANK/TRAF6 axis augments the phosphorylation of ERK, leading to the transcription of genes necessary for osteoclast differentiation and function, i.e., c-Fos, Atp6v0d2, RANK, and TRAP, and finally derive the differentiation of RAW 264.7 cell lines into osteoclasts (Kim B. et al., 2018).

As previously mentioned, in osteoporosis, the delicate balance of osteoblast-induced bone formation and osteoclast-induced resorption is compromised, leading to reduced bone mass and increased fragility. Similar to NF-κB, the overactivation of TRAF6 and subsequent MAPK signaling pathways can result in excessive osteoclastogenesis and bone resorption. For instance, augmented levels of pro-inflammatory cytokines like TNF-α and RANKL in osteoporosis patients enhance TRAF6 and MAPK activation, promoting osteoclast activity and leading to bone loss. The studies outlined have already firmly indicated that JNK is another serine/threonine protein kinase critical for maintaining osteoclast differentiation and proliferation. In this context, a recent study suggested that RANK and RANKL trigger cytoplasmic interactions by forming a complex with TRAF6 that activates TAK-1. The activation of TAK-1 leads to JNK phosphorylation, which significantly enhances osteoclast differentiation and proliferation from the RAW 264.7 cell line and BMMs. This enhancement is caused by the upregulation of DCSTAMP, Calcr, cathepsin K, TRAF-6, ACP5, NFATc-1, c-Fos, and V-ATPase d2 mRNA (Zhu et al., 2019; Huang et al., 2022). In more detail, phosphorylation of JNK facilitates the activity of the transcription factor c-Jun. Along with c-Fos, another crucial transcription factor involved in osteoclastogenesis, c-Jun forms the transcription factor complex known as AP-1. This complex attaches to the NFATc1 promoter, thereby regulating its expression. NFATc1 orchestrates the transcriptional regulation in osteoclasts, reputed for its ability to self-amplify to sustain high levels of expression, playing a critical role in osteoclast formation and function (Liu et al., 2021). Moreover, regulating TRAFs and MAPKs is crucial in maintaining bone homeostasis. Negative regulators, such as A20 and CYLD, can deubiquitinate and inactivate TRAF6, thereby attenuating MAPK signaling and osteoclast formation. Dysregulation of these harmful feedback mechanisms can further exacerbate osteoclast-mediated bone resorption in osteoporosis (Supplementary Table 1). Therapeutically, targeting the TRAF-MAPK pathway holds potential in treating osteoporosis. Inhibitors of RANKL, such as denosumab, have already shown efficacy in reducing osteoclast activity and bone resorption. Specific inhibitors of MAPKs or modulators of TRAF6 activity could also provide new avenues for preventing excessive bone loss by dampening the overactive signaling pathways (Shangguan et al., 2024).

In conclusion, the interaction between TNF receptor-associated factors and MAPKs is pivotal in governing the processes of osteoclastogenesis and bone resorption. A detailed understanding of this pathway elucidates the mechanisms leading to osteoporosis and highlights potential therapeutic targets for mitigating bone loss and improving bone health.

### 5.3 PI3K/AKT pathway: TRAF regulation of osteoclast and osteoblast balance

The PI3K/AKT pathway is another crucial signaling cascade involved in numerous cellular processes such as growth, survival, and metabolism. The interaction between TRAFs and the PI3K/AKT pathway has significant implications in the pathogenesis of osteoporosis. The PI3K/AKT pathway, on the other hand, is activated by various extracellular signals, including growth factors and cytokines. PI3K phosphorylates phosphatidylinositol (4,5)-bisphosphate (PIP2) to produce phosphatidylinositol (3,4,5)-trisphosphate (PIP3), leading to the activation of AKT. Activated AKT then phosphorylates a wide array of substrates engaged in cell survival, growth, and metabolic processes (Lin et al., 2024; Xi et al., 2015).

In the context of osteoporosis, the interaction between TRAFs and the PI3K/AKT pathway is particularly significant. TRAF6, for instance, is a crucial mediator of RANKL signaling in osteoclasts. Upon RANKL binding to its receptor RANK, TRAF6 is recruited to the receptor complex, activating downstream signaling pathways, like NF-κB and MAPK. Additionally, TRAF6 can stimulate the PI3K/AKT pathway, contributing to osteoclast survival and differentiation. This dual role of TRAF6 in activating both NF-κB/MAPK and PI3K/AKT pathways underscores its pivotal role in osteoclastogenesis and bone resorption.

Among the downstream regulators of AKT, glycogen synthase kinase-3 beta (GSK3β), a versatile serine/threonine kinase initially recognized for its role in regulating glycogen metabolism, has been found to exert the osteoclastogenesis effect of the TRAF6/PI3K/AKT pathway. When RANKL and M-CSF bind to their respective receptors, they initiate the activation of the PI3K/AKT signaling pathways, which are crucial for cell survival. AKT, once phosphorylated, initiates activation of downstream PI3K/AKT cascades, promoting osteoclast differentiation through the subsequent activation of the GSK3β/NFATc1 pathway necessary for osteoclast maturation. Similarly, TRAF- and PI3K/AKT-mediated transcription of NFATc1 increases the expression of osteoclast-specific genes comprising TRAP, DC-STAMP, CTR, V-ATPase d2, cathepsin K, and MMP-9, thereby enhancing both the differentiation and function of osteoclasts (Qu et al., 2021).

ROS significantly contributes to age-related osteoporosis through fostering the formation of osteoclasts and bone resorption. Regarding this aspect, it has been discovered that NOX1, a homolog of the catalytic subunit of the transmembrane enzyme NADPH oxidase-producing superoxide, plays an imperative role in the TRAF6/c-Src/PI3K axis-mediated stimulation of BMM differentiation into osteoclasts (Zhou et al., 2020). In fact, to induce the production of ROS, NOX1 should be activated in conjunction with GTP-Rac1. Moreover, Nox1 knockdown mitigated the quantity of osteoclasts produced by RANKL induction as well as the ROS generation caused by RANKL (Lee et al., 2005). Zhou et al. showed that RANKL elevated TRAF6 expression, activates c-Src, and promotes PI3K phosphorylation. This suggests that RANKL’s upregulation of Nox1 may be reliant on stimulation of the TRAF6–cSrc–PI3k signal, which in turn promotes the genesis of osteoclasts from BMMs by increasing ROS production (Zhou et al., 2020). The role of the TRAF6/PI3K/AKT cascade in ROS-related osteoporosis was more pronounced when a recent study validated the impact of this cascade on the transcription of malondialdehyde (MDA), superoxide dismutase (SOD), and glutathione (GSH) (Ma et al., 2018). MDA is a terminal byproduct generated during the peroxidation of polyunsaturated fatty acids within cellular environments. Elevated free radical concentrations drive excessive synthesis of MDA. Malondialdehyde levels are broadly acknowledged as a marker of oxidative stress and is used to assess the antioxidant status in patients (Cordiano et al., 2023). SOD serves as an antioxidant by rapidly eliminating oxygen radicals via redox cycles that engage transition metal ions at its active site. By doing so, SOD effectively prevents the formation of high ROS, including hydroxyl radicals and peroxynitrite (Zheng et al., 2023). Similar to SOD, GSH serves directly as an antioxidant, shielding cells from free radicals and pro-oxidants. Additionally, it acts as a cofactor for various antioxidant and detoxification enzymes, including glyoxalase and glutathione S-transferases (Chen et al., 2024). In this rationale, the study by Ma et al. indicated that stimulated TRAF6/PI3K/AKT signaling in ovariectomized rats is associated with overexpressed MDA and suppressed SOD and GSH, which subsequently encourages osteoclastogenesis and causes bone resorption as well as osteoporosis (Ma et al., 2018).

An overview of the literature indicates that among the signaling pathways, PI3K/AKT primarily interacts with NF-κB to influence the progression of osteoporosis. It has been implied that natural compounds such as *Cynomorium songaricum* and syringin prevent bone loss by blocking the action of the RANKL/RANK/TRAF6-mediated activation of NF-κB and PI3K/AKT pathways. This inhibition subsequently restrains osteoclastogenesis and prevents bone resorption, potentially offering therapeutic benefits against osteoporosis (Ma et al., 2018; Liu J. et al., 2018). This finding gave rise to the notion that targeting both of these pathways through the RANKL/RANK/TRAF6 axis could be a practical approach toward improvement in high-grade osteoporosis patients and also help develop novel targeted therapies.

Recent findings proposed that the PI3K lipid kinase has been abolished in modulating remodeling bone homeostasis by the specific activity of their phosphoinositides (PIs). PIs function as precise membrane docking locations for various signaling cascade effectors, in addition to being crucial as membrane constituents in eukaryotes and as precursors of second messengers like IP3 and PIP3. PIs, particularly PIP2, PIP3, and IP3, have been verified to play a number of crucial functions in both preserving bone homeostasis and osteoclast differentiation (Hammond and Burke, 2020). PIP5k1β is a member of the type 1 phosphatidylinositol 4-phosphate 5-kinase (PIP5k1) family, which includes the α, β, and γ isoenzymes, using phosphatidylinositol 4-phosphate as a substrate to synthesize phosphatidylinositol 4,5-bisphosphate [PI(4,5)P2]. It has been discovered that PIP5k1β was strongly expressed during the development of osteoclasts driven by RANKL and that PIP5k1β deletion led to bone loss in mice. Gain- and loss-of-function studies were conducted to examine the function of PIP5k1β in osteoblast and osteoclast differentiation, with the aim of gaining a deeper understanding of its role in bone homeostasis. PIP5k1β has the ability to suppress BMM transit and proliferation, which prevents osteoclast development. Furthermore, the deletion of PIP5k1β accelerated the growth and activity of osteoclasts by promoting the stimulation of MAPK and Akt signaling networks arising from the interaction between RANKL and its receptor RANK, which recruits TRAF6, ultimately allowing the expression of c-Fos, NFATC1 nuclear translocation, and osteoclastogenesis condition (Zhao et al., 2020). In osteoporotic conditions, the dysregulation of these pathways can lead to enhanced osteoclast activity and decreased osteoblast function, tipping the balance toward bone resorption. For example, elevated levels of pro-inflammatory cytokines like TNF-α in osteoporosis can enhance TRAF6 signaling, triggering the increased activity of NF-κB and PI3K/AKT pathways in osteoclasts, thereby promoting osteoclastogenesis and bone resorption (Xu et al., 2023).

Targeting the interaction between TRAFs and the PI3K/AKT pathway therapeutically presents a potential strategy for treating osteoporosis. Inhibitors of PI3K or AKT and modulators of TRAF6 activity are being explored for their potential to reduce excessive bone resorption and promote bone formation. Understanding the precise molecular mechanisms by which TRAFs and the PI3K/AKT pathway interact will be crucial in developing targeted therapies for osteoporosis and palliating bone health outcomes. In summary, the interaction between TRAFs and the PI3K/AKT pathway significantly regulates bone remodeling processes. Dysregulation of these pathways is attributed to the pathogenesis of osteoporosis by enhancing osteoclast activity and reducing osteoblast function. Therapeutic strategies targeting these pathways hold promise for the treatment and prevention of osteoporosis.

### 5.4 The ubiquitin-proteasome system: TRAFs in protein degradation and osteoclast regulation

The ubiquitin-proteasome system (UPS) plays a pivotal role in maintaining intracellular protein homeostasis and function, acting as a critical mechanism in protein degradation during bone formation or osteoclast differentiation. Dysregulation of ubiquitination processes can result in abnormalities in osteoclastogenesis, thereby disrupting bone resorption and potentially contributing to the development of osteoporosis (Fan et al., 2024). The UPS constitutes the primary pathway for protein degradation in eukaryotic cells and facilitates posttranslational modifications critical for protein metabolism, localization, regulation, and degradation, thereby ensuring protein homeostasis. Protein ubiquitination is executed through a tripartite mechanism: ubiquitin activation by the E1 enzyme, its transfer to the ubiquitin-conjugating enzyme E2, and subsequently, recognition of the target protein facilitated by E3 ubiquitin ligases. The E3 ligase then mediates the transfer of ubiquitin or a polyubiquitin chain from E2 to the substrate’s lysine residue or the protein’s N-terminal residue, which defines the substrate specificity of various ubiquitin ligases (Kleiger and Mayor, 2014).

Among the E3 ligases, the carboxyl terminus of the Hsp70-interacting protein (CHIP or STUB1) and Itch have received much attention due to their role in TRAF regulation of osteoporosis (Li et al., 2014; Zhang et al., 2013). CHIP, a protein containing a U-box domain, interacts with Hsp70 and acts as an E3 ligase for various protein substrates. It facilitates the ubiquitination and subsequent proteasome-dependent degradation of proteins such as p53, ER-α, Runx2, Src-3, and TLR4. CHIP is recognized for its critical roles in immunology, skeletal growth, and bone remodeling (Kumar S. et al., 2022). TRAF6 has been identified as a novel substrate of CHIP, potentially influencing osteoclastogenesis. To verify CHIP’s impact on TRAF6 protein levels, Li et al. conducted a Western blot analysis, revealing that increased CHIP expression in 293T cells corresponded with reduced TRAF6 protein levels. On the contrary, a mutant form of CHIP lacking E3 ligase activity did not affect TRAF6 protein levels. Additionally, in a parallel experiment, the reduction in TRAF6 protein levels was partially reversed by the proteasome inhibitor MG132, indicating that CHIP-mediated degradation of TRAF6 relies on the proteasome system. Moreover, ubiquitinated TRAF6 was immunoprecipitated using anti-ubiquitin antibodies from lysates of wild-type cells. However, TRAF6 ubiquitination showed a decrease in Chip−/− cells, implying that CHIP is involved in mediating TRAF6 ubiquitination. Their further analysis shows that since CHIP interacts with TRAF6 and increases its ubiquitin–proteasome degradation, diminishing the phosphorylation of IKK-β/I-κBα and impeding p65 translocation into the nucleus consequently, this cascade suppresses the NF-κB signaling pathway in osteoclasts (Li et al., 2014).

Like CHIP, Itch is another ubiquitin E3 ligase that regulates protein stability. Itch−/− mice exhibit a phenotype characterized by the development of progressive autoimmune disease. Additionally, patients with Itch mutations present autoimmune inflammatory cell infiltration in diverse tissues and complications in bone remodeling (Moser and Oliver, 2019). Parallel to ubiquitination, deubiquitination is another critical post-translational mechanism governing protein stability and cellular function. This process is orchestrated by deubiquitinating enzymes (DUBs), a class of proteases harboring DUB domains. Extensive genomic and functional analyses of the human genome have identified 95 DUBs, including notable examples such as A20 and cylindromatosis (CYLD) (Estavoyer et al., 2022). Notably, Itch lacks a DUB domain, suggesting a lack of intrinsic DUB activity. However, Itch interacts with CYLD and A20, implying its potential involvement in modulating protein deubiquitination via these DUBs (Nijman et al., 2005). In a recent investigation, Itch−/− mice displayed elevated osteoclast numbers and heightened responsiveness to lipopolysaccharide (LPS)-induced osteoclast formation and bone resorption. Notably, osteoclast precursors derived from Itch−/− mice exhibited prolonged RANKL-induced NF-κB activation and significant delays in TRAF6 deubiquitination following RANKL withdrawal. Further elucidating this phenomenon, Itch was found to restrict TRAF6 deubiquitination by binding to the deubiquitinating enzyme CYLD. In the absence of Itch, RANKL-induced NF-κB activation persisted, indicating a crucial role for Itch-mediated TRAF6 deubiquitination in regulating NF-κB signaling. These findings shed light on a novel mechanism whereby Itch-regulated TRAF6 deubiquitination contributes to persistent NF-κB activation in immune cells, potentially accounting for autoimmune phenotypes. Furthermore, targeting this pathway could represent a promising therapeutic strategy for mitigating osteoclast-mediated bone loss implications (Zhang et al., 2013). Considering the interaction of the deubiquitinating enzyme CYLD and TRAF6, it has been authenticated that the expression pattern of CYLD is similar to that of p62, a signaling adapter involved in both promoting and inhibiting osteoclastogenesis. p62 associates physically with TRAF6 and seems to enhance RANK signaling by recruiting atypical PKCs to TRAF6. When overexpressed, p62 also stimulates the self-ubiquitination of TRAF6, although the physiological significance of this remains unclear. The mechanisms by which p62 negatively regulates RANK signaling have not been well understood, although it is known that this inhibitory function requires the C-terminal region of p62. It has been demonstrated that p62 interacts directly with CYLD and facilitates the binding of CYLD to TRAF6. CYLD is incorporated into the TRAF6 complex, where it plays a crucial role in abating excessive TRAF6 ubiquitination in preosteoclasts. Furthermore, the C-terminal region of p62 is required for its role as an adapter in increasing CYLD/TRAF6 interaction. These results highlight a significant molecular interaction between p62 and CYLD and provide insights into the negative regulation of RANK signaling by p62. In fact, genes involved in osteoclastogenesis are induced when RANKL binds to RANK, leading to TRAF6 ubiquitination and activation of downstream signaling pathways. In addition to p62, CYLD expression is also upregulated as a consequence of RANK signaling. Assisted by p62, CYLD as a negative regulator of osteoclastogenesis, targets TRAF6, subsequently inhibiting TRAF6 ubiquitination and RANK signaling (Jin et al., 2008). It gets more confusing as CYLD may also serve as a promoter of osteoclastogenesis by regulating TRAF6 in another way through engaging the B-cell chronic lymphatic leukemia protein 3 (BCL3) as a partner protein. BCL3, identified as an interacting partner of TRAF6, regulates non-canonical I-κB signaling independently of IKK. Although the role of BCL3 in osteoclastogenesis remains unclear, evidence suggests that bone marrow-derived macrophages (BMDMs) lacking BCL3 have an increased propensity to differentiate into osteoclasts. CYLD works as an adapter to connect BCL3 to TRAF6, allowing TRAF6 to interact with BCL3. Upon RANKL stimulation, cylindromatosis (CYLD) is recruited to the TRAF6 complex. CYLD then removes the K63-linked polyubiquitin chain from BCL3, resulting in BCL3 retention in the cytoplasm. This retention inhibits the transcription of NF-κB target genes, including cyclin D1, and promptly facilitates the expression of osteoclastogenic markers like MMP-9, cathepsin K, NFATC1, and TRAP (Wang K. et al., 2018).

Fascinatingly, novel approaches are being suggested using USPs to provide a novel target for reversing excessive osteoclast formation. Deubiquitinating enzymes (DUBs) cleave ubiquitin from targeted proteins and ubiquitins in polyubiquitin chains, playing crucial roles in cell differentiation and organ homeostasis (Snyder and Silva, 2021). Recent research has shown that ubiquitin-specific protease 7 (USP7) is significantly upregulated in bone marrow macrophages (BMMs) following RANKL stimulation. Furthermore, knockdown or inhibition of USP7 has accelerated osteoclast formation and consequent bone resorption. In BMMs, USP7 acts as a deubiquitinating enzyme that inhibits osteoclastogenesis in a hybrid mechanism. This regulation occurs through the mediation of K63-linked ubiquitination of TRAF6 at its RING and coiled-coil (CC) domains. Additionally, USP7 facilitates the degradation of STING, which in turn enhances the expression of IFN-β (Xie et al., 2023). In contrast to the negative role of USP7 in bone resorption, USP25 was positively correlated with osteoclast differentiation based on menopausal circumstances. The study by Shen and colleagues indicated that USP25 controls the residual free TRAF6 in premenopausal women and encourages monocytes to differentiate into osteoclasts. On the other hand, TRAF6 is highly released in postmenopausal women due to the absence of estrogen, which in turn stimulates the overexpression of USP25 and eventually promotes osteoclast development. Nevertheless, USP25 had no effect on osteoclast differentiation in these postmenopausal women (Shen et al., 2022). Thus, novel therapeutic approaches could involve the inhibition of USP7 to prevent its negative regulation of osteoclastogenesis or the modulation of USP25 activity based on the menopausal status to control osteoclast differentiation.

With the deepening of the understanding of UPS’s role in maintaining protein homeostasis and regulating key signaling pathways in osteoclastogenesis, new avenues for treating bone-related disorders such as osteoporosis are emerging. One promising direction is the development of targeted therapies that modulate the activity of specific E3 ligases and DUBs involved in bone remodeling. Furthermore, advancements in gene editing technologies could facilitate precise manipulation of genes encoding particular components of the UPS, allowing for the development of gene therapies that correct dysregulated ubiquitination processes in osteoclasts. Combined with an improved understanding of the molecular mechanisms underlying UPS regulation and its close interaction with TRAFs in bone cells, these innovative approaches hold the potential to revolutionize the treatment of osteoporosis and related conditions, reducing the burden of bone diseases and improving the quality of life for affected individuals.

### 5.5 Keap1/Nrf2 pathway: oxidative stress regulation in bone metabolism

Nuclear factor erythroid 2-related factor 2 (Nrf2) is a ubiquitously expressed transcription factor that is critical in regulating genes involved in antioxidant defense, which preserves cellular redox balance and guards against oxidative stress. During oxidative stress or electrophilic conditions, the activity of Kelch-like ECH-associated protein 1 (Keap1) is inhibited, leading to Nrf2 release. Subsequently, Nrf2 translocates to the nucleus, where it binds to antioxidant response elements (AREs) within the promoters of genes encoding antioxidant enzymes, thereby safeguarding cells from reactive oxygen species (ROS). In the absence of stress, Nrf2 is sequestered by Keap1, which functions as an adapter for E3 ubiquitin ligase, facilitating Nrf2 degradation via the 26S proteasome pathway (Culletta et al., 2024; Suzuki et al., 2023). Accumulating data have displayed that the Nrf2 activator inhibits osteoclast development and hinders the development of osteoporosis, whereas Nrf2 itself reverses osteoclastogenesis and bone loss. The deficiency of Nrf2 results in enhanced RANKL-mediated osteoclast differentiation, MAPK signaling activation, and bone resorption due to elevated oxidative stress, which was partly attributed to the dysregulated production of antioxidant enzymes such as Srx, NQO1, and GSH (Hyeon et al., 2013). In the case of Nrf2 activators, RTA-408, a synthetic oleanane triterpenoid substance, has a significant cytoprotective influence relying on its ability to stimulate the Nrf2 signaling. By triggering the activity of Nrf2, RTA-408 suppresses the Lys63 polyubiquitination of STING by inhibiting the association between TRAF6 and STING, which in turn lessens the NF-κB signaling activity and consequent NFATC1-related osteoclast differentiation (Sun X. et al., 2020).

### 5.6 Non-coding RNAs: epigenetic regulation of TRAFs in osteoporosis

Noncoding RNAs (ncRNAs) constitute a category of RNA molecules that do not code for proteins. This group primarily includes microRNAs (miRNAs), long non-coding RNAs (lncRNAs), and circular RNAs (circRNAs). They influence the onset and progression of osteoporosis (OP) by modulating the target gene expression and epigenetic mechanisms. Recent research has revealed that some ncRNAs can be translated into peptides or small proteins. Specifically, certain ncRNAs encode functional peptides via their small open reading frames, participating in cellular signaling and gene expression regulation, thereby impacting the functions of osteoblasts (Han et al., 2024; Ebrahimnezhad et al., 2024). Among ncRNAs, identifying measurable biomarkers for both primary and secondary osteoporosis, such as miRNAs, is a vital strategy for risk evaluation. MiRNAs are small, single-stranded, non-coding RNA molecules, typically ranging from 18 to 24 nucleotides. They play a critical role in regulating mRNA expression by binding to the 3′-untranslated region (3′-UTR) of target mRNAs, thus modulating gene expression. MiRNAs are deeply involved in numerous biological processes, including cell proliferation, differentiation, development, and the regulation of bone homeostasis. Recent findings gave rise to the notion that some miRNAs comprising miR-181a-5p, miR-221-5p, miR-363-3p, and miRNA-146a may be implicated in osteoporosis by influencing TRAFs activity.

An essential role for miR-181a-5p is in the regulation of osteoporosis and inflammation. By targeting the NF-κB essential modulator (NEMO) and transforming growth factor β-activated kinase one-binding protein 2 (TAB2) to reduce blood vessel inflammation and NF-κB stimulation, mir-181a-5p mimic hampers the formation of atherosclerosis (Chen et al., 2018a). Further studies demonstrated that upregulation of miR-181a expression can promote apoptosis in osteoclasts induced by bone marrow-derived mesenchymal stem cells (BMMSCs), suppress osteoclastogenesis, and reduce bone resorption. Xue et al. (2023) found that employing a miR-181a-5p mimic led to elevated levels of miR-181a-5p, which inhibited the TRAF6/TAK1 signaling pathway and osteoclast formation. Additionally, it resulted in decreased levels of p-IKB-α and NF-kB65, reduced inflammation in RAW 264.7 cells, and attenuated bone resorption.

Despite the direct effect of miRNAs on the activity of TRAFs, some miRNAs may recruit interplay proteins to exert their function in bone remodeling. The SMAD3 protein operates within the TGF-β pathway, facilitating the transmission of signals from the cell membrane to the nucleus. This process is crucial for regulating gene expression and cell proliferation. Overexpression of Smad3 has been shown to enhance osteoclastogenesis, indicated by elevated levels of NFATc1 and TRAF6, an increased number of osteoclasts, and heightened cellular activity in RANKL-stimulated RAW 264.7 cells. It was hypothesized that Smad3 functions downstream of miR-221-5p in the regulation of osteoclastogenesis. To test this hypothesis, the dual-luciferase reporter assay subsequently confirmed that Smad3 is a direct target gene of miR-221-5p. Furthermore, *in vivo* experiments confirmed that mice subjected to ovariectomy (OVX) and injected with agomiR-221-5p exhibited decreased levels of Smad3, TRAF6, and NFATc1 and reduced osteoclast numbers. These findings indicate that miR-221-5p inhibits osteoclastogenesis by suppressing the Smad3/TRAF6/NFATc1 signaling cascades (Guo et al., 2022).

Except for the miRNAs, some other ncRNAs, like lncRNAs, might be involved in the competing endogenous RNA (ceRNA) network of the intricate TRAF-related osteoporosis mechanism. In various pathological conditions, lncRNA SNHG16 acts as a competing endogenous RNA (ceRNA) by sequestering microRNAs, notably miRNA-146a, decreasing miRNA-146a levels (Liu L. et al., 2018; Yang and Wei, 2019). Research has highlighted the critical functions of miRNA-146a in modulating inflammatory responses and oxidative stress. miRNA-146a plays a significant role in the onset and progression of numerous autoimmune diseases, such as arthritis, by specifically diminishing the production of inflammatory cytokines through the inhibition of TRAF6 (Tan et al., 2017; Nakasa et al., 2011). The interaction of miR-146a with the 3′-UTR of TRAF6 mRNA leads to the degradation of TRAF6 mRNA and a subsequent decrease in TRAF6 protein levels, thereby reducing the expression of related genes. It seems that SNHG16 may sponge miRNA-146a to elevate TRAF6 levels, influencing bone by upregulating SNHG16 expression, which in turn further increases TRAF6 levels. This elevation in TRAF6 significantly upregulates RANK expression while downregulating osteoprotegerin (OPG) expression in bone. This phenomenon was evidenced by CT scans, which demonstrated reduced bone density in male Balb/c mice (Helmy et al., 2024). Knockdown of lncRNA-Gm5532 hinders osteoclastogenesis and bone resorption. Similar to the mentioned research, other mechanistic studies indicate that lncRNA-Gm5532 operates as a ceRNA, acting as a sponge for miR-125a-3p, thereby enhancing the expression of TRAF6 and expressions of genes associated with bone resorption, comprising Car2, V-ATPase, CTSK, and MMP9 (Zhang et al., 2024c).

The intricate roles of ncRNAs in regulating bone homeostasis and osteoclastogenesis present novel opportunities for developing prognostic biomarkers, diagnostic tools, and therapeutic strategies for osteoporosis. Therefore, future research should focus on validating these ncRNAs’ clinical applications, their influence on TRAF signaling, and developing targeted therapies to manage and treat osteoporosis effectively.

### 5.7 TRAFs and TGF-β/BMP signaling: implications for osteoblast differentiation

TGF-beta plays a critical role in bone formation and remodeling that promotes the early differentiation of osteoblasts but inhibits their later differentiation into osteocytes. In a finely tuned microenvironment, TGF-beta has the potential to enhance the osteogenic differentiation of hMSCs while simultaneously suppressing their adipogenic differentiation (Du et al., 2021). In aging, mesenchymal progenitor cells (MPCs) exhibit elevated RANKL expression, leading to TRAF3 ubiquitination and its subsequent lysosomal degradation in osteoclast precursors (OCPs). This action stimulates osteoclastogenesis and bone resorption via the NF-κB pathway. Consequently, higher levels of TGFβ are released from the bone matrix and activated in the acidic milieu of resorption lacunae. The activated TGFβ further induces TRAF3 ubiquitination and degradation in MPCs, activating both RelA and RelB, enhancing RANKL production and bone resorption. Additionally, TRAF3 in MPCs interacts with TGFβR, negatively regulating GSK-3β activity to prevent β-catenin degradation. This leads to β-catenin accumulation and nuclear translocation, crucial for maintaining osteoblast differentiation and OPG secretion, which inhibits osteoclast formation. Ultimately, TGFβ1 mediates TRAF3 degradation and phosphorylates Tyr216, activating GSK-3β, which results in β-catenin degradation. This inhibition of osteoblast differentiation and OPG secretion, combined with increased RANKL expression, promotes osteoclast formation and bone loss (Li et al., 2019).

### 5.8 Klotho and age-related bone loss: a new perspective on TRAF signaling

Klotho is a 130-kDa transmembrane protein characterized by a short cytoplasmic tail; two extracellular domains, Kl1 and Kl2; and a transmembrane segment. It is predominantly found in the parathyroid glands and kidneys (Hu et al., 2016). In humans, the Klotho gene, comprising four introns and five exons, is situated on chromosome 5, flanked by the STARD13 and PDS5B genes, and shares homology with the mouse and rat genomes. The expression of Klotho is reduced under pro-inflammatory conditions, NF-κB signaling angiotensin II exposure, diabetic nephropathy, and matrix metalloproteinase activity. On the contrary, the PPAR-γ signaling, vitamin D, and several medications, including losartan, rapamycin, fosinopril, and statins, enhance Klotho expression. Significantly, klotho mutant (kL/kL) mice bearing hypomorph klotho alleles experience a condition characterized by traits associated with human aging, such as shortened life spans (3–4 months), aberrant mineral metabolism, and degradation of the nervous system. Osteoporosis is a vital characteristic of the aging-related phenotypes observed in Klotho mutant mice (Kanbay et al., 2024; Kawaguchi et al., 2000). An intriguing discovery in kl/kl mice is that low-turnover osteoporosis results from impairments to osteoblasts and osteoclasts; it does not rely on interactions between them. Klotho has been described to mediate RANKL-induced osteoclastogenesis. When RANK and Klotho are linked, the RANK and TRAF6 interaction was enhanced in osteoclastic cells administered RANKL. This led to the triggering of the downstream NF-kB signaling cascade, which in turn increased the transcription levels of c-Fos and NFATc1. Consequently, Klotho may serve as a promising therapeutic option for addressing osteopenic disorders associated with osteoclast activity (Yu et al., 2021).

### 5.9 The immune system and inflammatory cytokines: TRAF modulation of osteoimmunology

The immune system’s interconnectedness and bone loss have been acknowledged for centuries, although the recognition of a functional relationship has emerged only in recent years. The immune system’s involvement in the onset of senile osteoporosis, primarily driven by estrogen deficiency and secondary hyperparathyroidism, is gradually being elucidated. Immune cells, various cytokines, and signaling pathways contribute significantly to the bone remodeling process. The interaction between the skeletal and immune systems has given rise to the interdisciplinary field of osteoimmunology. Within this framework, the NF-κB receptor RANK/RANKL/OPG axis is crucial to bone remodeling and has been thoroughly studied. Additionally, the roles of other proinflammatory cytokines, including the TNF family and the IL family, have been extensively examined in the context of bone remodeling (Xu et al., 2023). Considerable evidence in the previous 2 decades entrenched ILs like as IL-1, IL-10, and IL-35 in the participation of osteoclastogenesis through modulation of TRAF-mediated signaling pathways (Li et al., 2005).

Li and colleagues’ investigation has provided strong validation for IL-1’s function in osteoclast development (Li et al., 2005). To facilitate a comprehensive understanding of this research, a definition of IL-1, interleukin-1 receptor-associated kinase (IRAK), and TLRs should be discussed. IL-1R-associated kinase (IRAK) is pivotal in the signaling pathways initiated by IL-1R/TLR family members, due to its conserved intracytoplasmic domain, “TLR- and IL-1R-related.” Consequently, the activation of various members of this extended family triggers comparable signaling cascades that ultimately result in the activation of MAPKs and the IκB kinase complex (Janssens and Beyaert, 2003; Kim et al., 2024). IRAKs are multidomain proteins characterized by a death domain and a conserved central kinase domain that facilitates an interaction with the myeloid differentiation factor 88 (MyD88). Although all IRAKs possess a functional ATP-binding site, IRAK-2 and IRAK-M are catalytically inactive. This inactivity arises because a crucial aspartate residue, essential for catalytic activity, is substituted by asparagine in IRAK-2 and by serine in IRAK-M. Consequently, IRAK-2 and IRAK-M lack kinase activity (Gürkan et al., 2024). IRAK-M functions as a suppressor of IL-1R and TLR signaling pathways by inhibiting the dissociation of other IRAK proteins from MyD88. This action helps maintain the integrity of the IRAK–TRAF6 complex within the TLR signaling pathway. By effectively trapping IRAKs within the receptor complex, IRAK-M functions to block the subsequent activation of NF-κB and MAPK signaling cascades downstream (Xu et al., 2023). Therefore, IRAK-M serves a crucial role as a dominant negative regulator in macrophages, acting downstream of IL-1R/TLR signaling pathways. The study by Li et al. (2005) reveals that IRAK-M plays a crucial role in regulating IL-1R signaling, promoting transient activation and survival of osteoclasts. Without IRAK-M, there is constitutive activation of IL-1R/TLR pathways, resulting in continuous IL-1 production and TRAF6-mediated activation of NF-κB correlating with prolonged osteoclast survival and activation. Therefore, IRAK-M, predominantly expressed in myeloid cells and strongly induced by RANKL, emerges as a pivotal regulator that effectively suppresses the differentiation of osteoclasts by influencing the interaction of IL-1 and TRAF6.

In contrast, the role of IL-1 and IL-35 in promoting osteoclastogenesis has been found to negatively regulate this process. A well-known study on RAW 264.7 macrophage cells revealed that IL-35, known for its anti-inflammatory properties, suppresses TNF-α-related osteoclast formation by abating the activity of the TRADD-TRAF2-NF-kB signaling pathway. Concurrently, it enhances the TRADD-FADD-caspase 3 pathway activation via JAK1/STAT1 signaling. It has been indicated that co-treatment with TNF-α and IL-35 leads to upregulation of TNFR1, FADD, and cleaved caspase 3. On the contrary, IL-35 attenuates TNF-α-induced increases in TRAF2 and RIP1, thereby palliating NF-κB DNA-binding activity. In addition, fludarabine, a selective p-STAT1 inhibitor, markedly reversed the effects of IL-35 on suppressing TNF-α-induced osteoclastogenesis and augmenting apoptosis, indicating that IL-35 impedes TNF-α-induced activation of the TRAF2/NF-κB pathway while triggering the FADD-caspase 3 apoptosis through JAK1/STAT1 signaling (Peng et al., 2018).

ILs present a promising therapeutic avenue for various inflammatory diseases such as osteoporosis, owing to their pivotal role in regulating TRAFs. Investigating the effects of IL-35 and IL-1 on human osteoclasts holds substantial translational potential in advancing therapeutic interventions in this context.

### 5.10 Smad

The interaction between TRAFs, particularly TRAF2, and Smad signaling has garnered attention in research on osteoporosis due to its impact on bone health, especially in osteoblast differentiation and bone formation. BMPs, part of the TGF-β superfamily, are critical in driving osteoblast maturation, both in experimental and *in vivo* contexts, making them essential to maintaining bone structure. Over 20 BMP-related proteins—including BMP-2, BMP-4, and BMP-7—bind to BMP type I and type II serine/threonine kinase receptors, which, upon activation, initiate phosphorylation cascades crucial for cellular signaling. BMP type II receptors activate BMP type I receptors via phosphorylation, which in turn stimulates receptor-regulated Smads (R-Smads) such as Smad1, Smad5, and Smad8, essential for bone development. This pathway contrasts with the activation of Smad2 and Smad3, which respond to activin and TGF-β signals via specific receptor interactions (Segredo-Morales et al., 2017; Zou et al., 2021).

Following phosphorylation, R-Smads form complexes with Smad4, the only Co-Smad in mammals, facilitating their entry into the nucleus to influence gene expression critical to osteoblast differentiation. BMP signaling triggers transcription factors like Runx2, Dlx5, Msx2, and osterix, which are key to the development and maturation of bone-forming cells. In osteoporosis, TRAF2 is thought to inhibit BMP signaling as TNF-α exposure results in decreased phospho-Smad1, BMPR-IA, and Runx2 levels, while BMPR-IB and BMPR-II levels increase. Notably, a short-term TNF-α stimulus combined with TRAF2 silencing enhances Smad1’s nuclear accumulation, a result not seen without TRAF2 knockdown. These findings imply that TRAF2 may block Smad1’s nuclear translocation, potentially through interactions with the NF-κB pathway, highlighting the regulatory role in osteoblast function via I-κB kinase activation, I-κB phosphorylation, and ubiquitination (Shimada et al., 2009).

In parallel, Smad signaling plays an essential role in osteoclastogenesis, especially in response to RANKL. Blocking TGF-β signaling with inhibitors like SB431542 or via mutant TGF-β receptors suppresses RANKL-induced osteoclast differentiation. Furthermore, the disruption of Smad signaling by overexpressing Smad7 or c-Ski significantly reduces RANKL-induced osteoclastogenesis. On the contrary, activating Smad2 or Smad3 can counteract these inhibitory effects, indicating a crucial role for Smad2/3 in osteoclast formation. Notably, Smad3 is directly associated with the TRAF6-TAB1-TAK1 complex, which is vital for RANKL-induced osteoclast differentiation. This interaction is essential for downstream signaling and is absent when TGF-β signaling is disrupted. The MH2 domain of Smad3 is required for TRAF6-TAB1-TAK1 complex formation and subsequent signaling in osteoclastogenesis, underscoring Smad3’s centrality in bone resorption processes (Yasui et al., 2011).

In summary, TRAF2 appears to modulate BMP signaling in osteoblasts, impacting Smad1 activity and bone formation, while Smad3’s interaction with TRAF6 is essential for osteoclast differentiation in response to RANKL. These pathways, interconnected by TRAF and Smad interactions, represent potential therapeutic targets for regulating bone metabolism in osteoporosis.

## 6 Therapeutic approach

### 6.1 Exploring natural products for TRAF targeting in osteoporosis management

Natural compounds, which often have fewer side effects than synthetic drugs, have demonstrated substantial efficacy in modulating this pathway, making them potent candidates for osteoporosis treatment (Supplementary Table 1) (Ilyas et al., 2024; Wang et al., 2023). Here, we discuss several natural products and their mechanisms in targeting TRAFs and RANKL/TRAF signaling, focusing on their effects on osteoclastogenesis, osteoblast differentiation, and bone resorption.

Sparganii Rhizoma (SR), derived from *Sparganium stoloniferum*, is traditionally used in Korean medicine for gynecological conditions and has recently been investigated for its effects on bone health. SR is rich in compounds like phenylpropanoids (e.g., ferulic acid, p-coumaric acid, and caffeic acid), flavonoids, and coumarins (Lee et al., 2021). These bioactive components have been shown to inhibit osteoclast differentiation by downregulating key signaling proteins in the RANKL-TRAF6 cascade, such as NFATc1 and c-Fos. SR’s anti-inflammatory properties further contribute to its anti-osteoporotic potential as it suppresses inflammatory cytokines TNF-α and IL-6, both of which are associated with bone resorption in osteoporosis. Studies using OVX (ovariectomized) models indicate that SR not only inhibits osteoclast differentiation but also promotes osteoblast activity through the BMP-2/SMAD pathway, underscoring its dual-action potential as an osteoclastogenesis inhibitor and an osteogenesis stimulant (Lee et al., 2021). Puerarin, an isoflavone glycoside from *Pueraria radix*, is recognized for its effects on reducing bone loss in a concentration-dependent manner. Puerarin inhibits RANKL signaling by promoting osteoprotegerin (OPG) expression, a decoy receptor for RANKL that prevents it from binding to its receptor on osteoclast precursors. Moreover, puerarin exerts antioxidant effects that reduce ROS (reactive oxygen species) levels in bone marrow, which are otherwise elevated by RANKL signaling. Through inhibiting ROS-induced MAPK and NF-κB pathways, puerarin downregulates osteoclast-specific transcription factors like NFATc1, thereby reducing osteoclast differentiation and activity. Comparative studies with other compounds suggest that puerarin’s distinct role in oxidative stress modulation is an added advantage, potentially making it an ideal candidate for postmenopausal osteoporosis, where ROS and inflammation levels are high (Xiao et al., 2020). Cytisine, a quinolizidine alkaloid from plants in the Leguminosae family, has shown promise in countering osteoporosis by inhibiting RANKL-induced osteoclastogenesis. Cytisine acts on various signaling pathways, including JNK/ERK/p38-MAPK and PI3K/AKT, reducing expressions of osteoclast markers such as NFATc1 and MMP-9. This compound’s ability to inhibit the PI3K–AKT–NFATc1 axis was found to be reversible with the addition of an AKT activator, indicating a specific suppression of osteoclast differentiation through this pathway. Cytisine directly impacts bone resorption by preventing F-actin ring formation and reducing TRAP activity in osteoclasts. Compared with other compounds, cytisine’s mechanism is notably robust in modulating the TRAF6–PI3K/AKT axis, making it effective in both RANKL-induced and RANKL-independent osteoclastogenic pathways (Qian et al., 2020; Wang et al., 2024). *Paris polyphylla* and its active component, polyphyllin VII have shown antioxidant and osteoprotective effects, particularly through modulation of intracellular ROS. Polyphyllin VII prevents the activation of Nox1, an NADPH oxidase critical for ROS production in osteoclast precursors, by blocking the TRAF6-cSrc-PI3K pathway. This inhibition of ROS generation subsequently reduces RANKL-induced osteoclast differentiation. Polyphyllin VII downregulates GTP-Rac1, further preventing Nox1 activity and decreasing oxidative stress in bone marrow cells. When compared with SR and puerarin, polyphyllin VII’s antioxidant capability is highly potent, especially in inhibiting the TRAF6-Nox1-mediated ROS pathway, which makes it particularly effective for oxidative stress-induced bone resorption common in osteoporosis (Zhou et al., 2020; Yang et al., 2023). Pristimerin, a triterpenoid from the Celastraceae family, offers another approach that targets inflammation and the RANKL pathway. It has been observed to restore TRAF6 expression and inhibit NF-κB activation in osteoclasts from OVX models, thereby decreasing osteoclast differentiation and bone resorption. Additionally, pristimerin reduces inflammatory cytokines and NF-κB translocation to the nucleus, modulating the inflammatory environment that promotes osteoporosis in estrogen-deficient states. Pristimerin’s regulatory effect on both PI3K and NF-κB pathways appears similar to that of SR, although its anti-inflammatory action is more pronounced, making it beneficial in inflammatory and estrogen-deficient osteoporosis (Xu et al., 2022).

**FIGURE 3:**
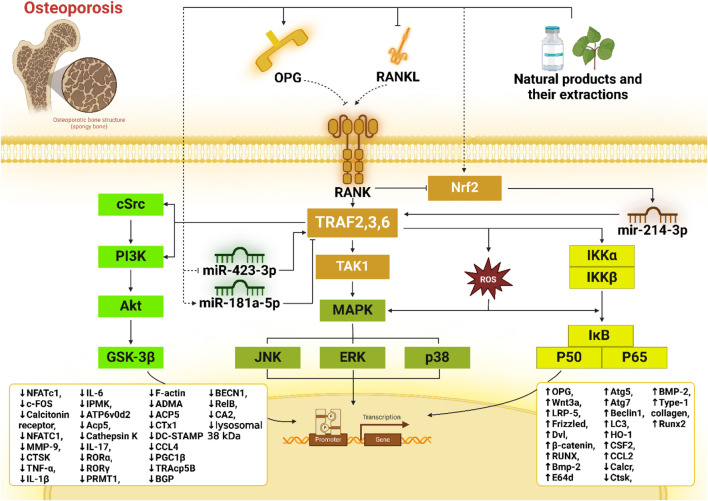
Schematic representation of the signaling pathways involved in osteoclast differentiation and osteoporosis activity highlights molecular targets for potential therapeutic intervention by natural products. The illustration shows the interaction between RANKL (receptor activator of nuclear factor kappa-Β ligand) and its receptor RANK, which activates downstream signaling cascades, including TRAF-mediated pathways (TRAF2, TRAF3, and TRAF6) and essential kinases (TAK1, MAPK, JNK, ERK, and p38). Natural compounds and their extracts influence these pathways, with effects on regulators such as NF-κB, Nrf2, and various microRNAs (e.g., miR-423-3p, miR-181a-5p, and miR-214-3p), ultimately modulating expressions of genes and proteins associated with osteoclastogenesis and bone resorption.

The natural compounds discussed above exhibit distinct yet overlapping mechanisms in modulating TRAF signaling pathways. SR and puerarin primarily enhance osteoblast differentiation and reduce osteoclastogenesis through BMP-2/SMAD and ROS pathways. Cytisine and polyphyllin VII target oxidative stress and inflammatory pathways mediated by RANKL/TRAF6 signaling, showcasing their potential as anti-osteoporotic agents through regulating oxidative and inflammatory responses. Meanwhile, pristimerin combines anti-inflammatory and anti-osteoclastogenic effects, positioning it as a potential alternative to synthetic drugs with fewer side effects. By targeting both upstream and downstream regulators in the RANK/TRAF6 pathway, these compounds inhibit osteoclast differentiation and promote osteoblast activity (Figure 3). This dual approach—modulating osteoclastic activity while enhancing osteogenic responses—illustrates the potential of natural compounds to holistically address osteoporosis. Future research could focus on optimal dosing regimens and exploring synergistic effects among these compounds to enhance the therapeutic efficacy and safety in osteoporosis management.

### 6.2 Polyphenols targeting TRAFs in osteoporosis

Osteoporosis is primarily characterized by excessive bone resorption, often driven by osteoclast overactivation, which is a consequence of increased RANKL signaling. Targeting this pathway has been instrumental in developing therapies that can mitigate bone loss by interfering with osteoclast formation and function. Polyphenols, a diverse group of plant-derived compounds, show great potential in modulating RANKL/TRAF signaling, thereby acting on pathways involved in osteoclastogenesis (Su et al., 2024). Notably, resveratrol (Res), curcumin, icariin, and xanthohumol each exhibit unique mechanisms that influence upstream, downstream, and intermediary regulatory molecules within the RANKL/TRAF signaling axis (Supplementary Table 1).

Res has shown promise in reducing osteoclast activity and enhancing osteoblast differentiation, addressing two significant aspects of bone homeostasis. In elderly patients’ bone marrow stromal cells (BMSCs), Res increased osteoblast differentiation while simultaneously decreasing adipogenesis, likely through the suppression of NF-κB signaling. Additionally, Res was found to inhibit TRAF6, a crucial adapter in RANKL-induced osteoclastogenesis, thereby attenuating the activation of downstream pathways like TAK1 and NF-κB. This inhibition reduces inflammation and osteoclast differentiation, helping limit bone resorption. Res also upregulates miR-181a-5p, a microRNA implicated in repressing TRAF6, demonstrating its potential to target RANKL/TRAF signaling and effectively modulate osteoclast function (Xue et al., 2023). Curcumenol (CUL), an antioxidant isolated from *Curcuma zedoaria*, has also shown efficacy in mitigating RANKL-induced osteoclastogenesis by promoting TRAF6 degradation via K48-linked polyubiquitination. This process leads to reduced activation of both MAPK and NF-κB pathways, ultimately decreasing osteoclast formation and bone resorption. Furthermore, CUL interferes with inositol polyphosphate multikinase (IPMK) binding to TRAF6, which, under normal conditions, enhances TRAF6 stability. CUL effectively impairs TRAF6 signaling by disrupting this association, reducing osteoclast activity and bone preservation in osteoporotic models. This degradation mechanism highlights a novel approach to inhibiting osteoclastogenesis by directly reducing the stability of a key RANKL signaling component (Wang S. et al., 2021).

Curcumin, another widely studied polyphenol, has a complex interaction with RANKL-induced osteoclastogenesis, partly through its regulation of autophagy. While curcumin promotes autophagy in osteoclast precursors (OCPs), this effect paradoxically supports both the proliferation and differentiation of OCPs. By upregulating Atg7 and Beclin1, curcumin promotes autophagy in OCPs, which can enhance osteoclast formation. However, when combined with autophagy inhibitors like chloroquine, curcumin’s anti-osteoclastogenic effects become more pronounced as the counteracting autophagy-mediated support for osteoclast differentiation is neutralized. This intricate relationship suggests that curcumin could be optimized for osteoporosis treatment alongside autophagy inhibitors, which may amplify its inhibitory effects on RANKL/TRAF signaling and osteoclastogenesis (Ke et al., 2020). Icariin, derived from *Epimedium* species, is known to inhibit RANKL-induced osteoclastogenesis through the downregulation of TRAF6 expression. By suppressing TRAF6, icariin also inhibits ERK and NF-κB activation, which are pivotal in osteoclast survival and resorption. The reduction of downstream markers like NFATc1, c-Fos, RANK, and TRAP compounds this effect. Additionally, icariin blocks the phosphorylation of ERK without affecting other MAPK pathway elements, highlighting its targeted impact on the RANKL/TRAF6 axis. This modulation reduces osteoclastogenesis, making icariin a potential candidate for osteoporotic treatment (Kim B. et al., 2018). Xanthohumol (XN), a prenylflavonoid from hops, has demonstrated a unique capacity to disrupt the RANK–TRAF6 interaction directly, thereby inhibiting the RANKL-induced activation of NF-κB and Ca2+/NFATc1 pathways. This interruption significantly reduces osteoclastogenesis and bone resorption *in vitro* and *in vivo*, as demonstrated in ovariectomy-induced osteoporosis models. XN’s ability to block TRAF6 association highlights a different mechanism by which polyphenols can interfere with osteoclast differentiation and function, providing a promising therapeutic approach for conditions characterized by pathological bone resorption (Li et al., 2015).

In conclusion, the distinct mechanisms by which these polyphenols interact with RANKL/TRAF signaling pathways showcase their therapeutic potential in treating osteoporosis. Res primarily acts by enhancing osteoblast differentiation and inhibiting TRAF6 through miR-181a-5p modulation, offering a dual approach to bone preservation. Curcumenol focuses on degrading TRAF6 and disrupting its interaction with IPMK, thereby reducing osteoclast activity effectively. Curcumin presents a more complex profile by promoting autophagy while simultaneously exerting anti-osteoclastogenic effects, which can be enhanced in conjunction with autophagy inhibitors. Icariin selectively downregulates TRAF6 and suppresses key signaling pathways critical for osteoclast survival, providing a focused strategy against osteoclastogenesis. Finally, xanthohumol directly disrupts the RANK–TRAF6 interaction, demonstrating a novel and effective method to inhibit osteoclast differentiation. Collectively, these polyphenols present a multifaceted approach to osteoporosis treatment, each contributing unique mechanisms that target the RANKL/TRAF signaling axis, paving the way for future therapeutic developments.

### 6.3 Nanoparticles targeting TRAFs in osteoporosis

In recent years, nanoparticles have emerged as a promising strategy for targeting TRAFs in osteoporosis, leveraging their unique properties to modulate cellular processes linked to bone remodeling. Specifically, inorganic nanoparticles, such as calcium, magnesium, zinc, iron, silicon, and strontium-based materials, have shown potential to mitigate bone degeneration by promoting osteoblast differentiation and mineralization (Wen et al., 2024; Hao et al., 2024). Hydroxyapatite nanoparticles (HANPs), for instance, have become a focal point in osteoporosis research due to their biomimetic composition and nanoscale size, which allow for integration with bone tissue, thereby enhancing osteogenesis through pathways such as Wnt/β-catenin, TGF-β/Smad, DNA methylation, and calcium homeostasis. In addition to fostering bone formation, these inorganic nanoparticles have demonstrated inhibitory effects on osteoclastogenesis, which is essential in counteracting bone resorption—a critical factor in osteoporosis progression (Zhao et al., 2017).

Recent advances have highlighted specific nanocomposites’ dual role in modulating osteoclast and osteoblast activities. Superparamagnetic iron oxide nanoparticles (SPIONs), coated with hydroxyapatite (SPIO@HA), have been synthesized in various Fe/Ca molar ratios. These SPIO@HA nanocomposites, particularly at a 1:15 Fe/Ca ratio, not only promote osteogenesis in mesenchymal stem cells (MSCs) but also suppress osteoclastogenesis by inhibiting RANKL-induced formation of TRAP-positive multinucleated cells and actin rings. SPIO@15HA displays enhanced retention in bone marrow compared to uncoated SPIOs, potentially leading to a more prolonged therapeutic effect, as shown by improved bone microstructure in ovariectomized mouse models, a common osteoporosis model. This dual function is partly mediated by the upregulation of p62, which downregulates TRAF6 activity—a key signaling molecule in RANKL-induced osteoclastogenesis. SPIO@HA’s ability to enhance bone formation while inhibiting bone resorption underscores its potential as a comprehensive treatment for osteoporosis (Li et al., 2021). Iron oxide nanoparticles (IONPs), commonly used as MRI contrast agents and drug carriers, have recently been found to have intrinsic therapeutic effects on bone health. With a high affinity for bone tissue, exceptionally when engineered at specific sizes, IONPs are readily internalized by bone marrow macrophages (BMMs), which are precursors to osteoclasts (Rahman, 2023). Studies indicate that IONPs, specifically ferumoxytol and ferucarbotran, can significantly attenuate osteoclastogenesis in osteoporosis by modulating the TRAF6 signaling pathway. IONPs upregulate p62 expression via activation of the TLR4–Nrf2 signaling axis, stabilizing the TRAF6–p62–CYLD complex, promoting deubiquitination of TRAF6. This modulation of TRAF6 reduces downstream activation of NF-κB and MAPK signaling pathways, effectively lowering the expression of osteoclast-related genes and thus suppressing bone resorption. Given their capacity to simultaneously serve diagnostic and therapeutic purposes, IONPs represent a multifunctional therapeutic option in osteoporosis (Liu et al., 2020). Emerging research on ultra-fine bubbles (UFBs), or nanobubbles, adds a novel dimension to nanoparticle-based interventions targeting TRAFs in bone disease. UFBs, particularly oxygen-enriched varieties, have demonstrated potential in enhancing local oxygenation, which can beneficially impact bone metabolism (Knowles et al., 2024). Hypoxic conditions within the bone marrow can accelerate osteoclastogenesis, exacerbating osteoporosis. UFBs improve oxygen availability, potentially reducing the activity of hypoxia-induced factors like HIF-1α, which promotes osteoclast differentiation. This oxygen-mediated modulation of the RANKL/TRAF6 pathway offers a unique approach to balance osteoclast and osteoblast activities, with UFBs downregulating TRAF6, c-Fos, and NFATc1 expressions, as well as phosphorylation of p38 MAPK in osteoclasts, without affecting oxygen-insensitive factors. Further research into the exact mechanisms of UFBs in bone tissue may reveal new therapeutic applications for hypoxia-related osteoporosis (Noguchi et al., 2017).

Overall, integrating nanoparticles targeting TRAFs and RANKL signaling in osteoporosis offers a multifaceted approach, influencing both upstream and downstream regulators in the bone remodeling process. By attenuating TRAF6 signaling and modulating the RANKL pathway, these nanoparticles hold promise in controlling osteoclastogenesis while fostering osteogenesis, marking a significant advancement in osteoporosis therapy.

### 6.4 Microbiomes targeting TRAFs in osteoporosis

The gut microbiome, often considered a central regulator of systemic health, exerts a substantial influence over the immune and skeletal systems, suggesting its potential as a therapeutic target for osteoporosis (Ahire et al., 2024). Research has revealed that metabolites produced by gut microbiota, such as trimethylamine-N-oxide (TMAO), may influence the RANKL/TRAF signaling pathways linked to osteoclastogenesis. TMAO is a metabolite derived from gut microbiota processing dietary L-carnitine and phosphatidylcholine-rich foods, including red meat, dairy, and eggs. After digestion, trimethylamine (TMA) is produced and converted to TMAO in the liver by flavin monooxygenase 3 (FMO3). Elevated TMAO levels have been correlated with increased levels of pro-inflammatory cytokines, ultimately contributing to various inflammatory conditions, including osteoporosis. Recent studies have highlighted that TMAO may contribute to bone loss by promoting osteoclast differentiation through the ROS-dependent NF-κB signaling pathway, as demonstrated by increased TRAP-positive osteoclast formation and the upregulation of vital osteoclast-related genes (e.g., TRAF6, c-Fos, and NFATc1). Inhibitors of NF-κB signaling and ROS, such as BAY 11–7082 and N-acetylcysteine (NAC), have been shown to reduce these TMAO-induced effects, suggesting potential therapeutic targets within the microbiome’s metabolic pathways (Wang N. et al., 2022).

Probiotics, precisely strains such as *L. brevis* AR281 and *Lactobacillus curvatus* Wikim 38 (LC38-CS), have demonstrated anti-osteoporotic effects through their anti-inflammatory properties and direct modulation of RANKL/TRAF pathways (Yu et al., 2022), (Jang et al., 2021). The RANKL/OPG/RANK system is essential for regulating osteoclast differentiation; RANKL binds to RANK, initiating downstream signaling cascades that lead to the activation of TRAF6, NF-κB, and ultimately NFATc1, a critical transcription factor for osteoclastogenesis. Probiotics like *Lactobacillus brevis* AR281 have been shown to attenuate the RANKL/OPG ratio, thereby reducing osteoclast formation. In addition, AR281 downregulated pro-inflammatory cytokines such as IL-1, IL-6, and IL-17, which are known to enhance osteoclast differentiation by upregulating RANKL and downregulating OPG, thus further supporting its anti-osteoclastogenic properties (Yu et al., 2022). Similarly, LC38-CS has exhibited a dose-dependent inhibition of RANKL-induced osteoclast differentiation and bone resorption in osteoporotic models. LC38-CS downregulates early RANKL signaling events, particularly within the TRAF6/NF-κB/MAPK axis, by suppressing the phosphorylation of NF-κB p65 and MAPK pathway proteins (ERK, p38, and JNK). This results in downregulated expression of osteoclast-related genes and markers such as PRMT1 and ADMA, which are novel biomarkers for osteoclastogenesis. Additionally, LC38-CS has been shown to improve bone mineral density (BMD) in ovariectomized mice, supporting its potential as a therapeutic candidate (Jang et al., 2021).

Although with contrasting effects, both TMAO and specific probiotics demonstrate an influential role in modulating osteoclastogenesis through RANKL/TRAF/NF-κB signaling. Although elevated TMAO levels drive inflammatory osteoclast activation, probiotic interventions can counteract osteoclast differentiation and bone loss by targeting RANKL/OPG balance and TRAF6 signaling. These findings underscore the potential of probiotic modulation of the gut microbiome as a therapeutic avenue for osteoporosis. Further studies focusing on gut microbiota-derived metabolites and probiotic strains could provide novel insights into developing microbiome-based treatments targeting TRAF-related pathways in bone disease.

### 6.5 Clinical trials

Recent clinical trials have begun exploring the efficacy of natural products, particularly those with bioactive compounds targeting TRAFs, for osteoporosis management. A notable study investigated Epimedium prenylflavonoids (EP), compounds derived from the plant Epimedium, known for their potential in osteoporosis treatment due to their ability to modulate TRAF6—a crucial mediator in osteoclastogenesis and bone resorption pathways. A randomized, double-blind, placebo-controlled clinical trial examined the safety and effects of EP prenylflavonoids in postmenopausal women over a 6-week period. Participants received either 740 mg of the EP extract daily or a placebo. The primary outcomes focused on safety and pharmacokinetics, as well as indicators of bone turnover, such as bone-specific alkaline phosphatase (BSAP) and TRAF6 levels in peripheral blood monocytes. The trial findings indicated that EP prenylflavonoid intake was safe, showing no significant adverse effects in hepatic, hematological, or renal functions. The primary metabolites detected in serum—desmethylicaritin and icaritin—were associated with increased levels of BSAP, suggesting an anabolic effect on bone formation. There was also a trend toward reduced TRAF6 levels in peripheral monocytes, indicating a potential suppression of osteoclast activity. This reduction in TRAF6 aligns with preclinical studies where EP components inhibited osteoclastogenesis by downregulating TRAF6, thereby limiting bone resorption (Yong et al., 2021).

These promising outcomes underscore EP’s dual role in enhancing bone formation while inhibiting bone resorption, making it a potential therapeutic candidate for osteoporosis. However, further studies are necessary to confirm these effects over longer durations and across diverse populations. This evidence supports the viability of TRAF-targeted natural therapies, presenting a safer, well-tolerated option that addresses both bone anabolic and anti-resorptive needs in osteoporosis treatment.

### 6.6 Strategies for targeted delivery of natural compounds in osteoporosis therapy

Natural compounds such as flavonoids, polyphenols, and alkaloids have gained significant attention due to their ability to modulate key signaling pathways involved in osteoblastogenesis and osteoclastogenesis. However, their clinical translation remains challenging due to poor water solubility, rapid metabolism, and systemic side effects. To overcome these limitations, innovative drug delivery systems have been developed to enhance the bioavailability and site-specific action of these compounds while minimizing adverse effects. Several strategies, including nanoparticle-based systems, liposomal formulations, bisphosphonate-functionalized drug conjugates, smart hydrogels, micelle-based systems, and prodrug strategies, have emerged as promising approaches for targeted osteoporosis therapy.

#### 6.6.1 Nanoparticle-based delivery systems

Nanotechnology has revolutionized the field of drug delivery by offering precise control over drug release, enhanced cellular uptake, and improved stability. Nanoparticles (NPs) serve as effective carriers for osteoprotective compounds by encapsulating bioactive molecules and directing them to bone tissues. Polymeric nanoparticles such as poly(lactic-co-glycolic acid) (PLGA) and chitosan have been widely explored for delivering phytochemicals like curcumin, resveratrol, and icariin, significantly enhancing their therapeutic efficacy (Ahn et al., 2017). Functionalization of nanoparticles with bone-seeking moieties such as alendronate or hydroxyapatite has been shown to improve drug accumulation in bone tissue, reducing systemic clearance and enhancing osteoprotective effects. For instance, PLGA nanoparticles conjugated with hydroxyapatite have demonstrated superior bone-targeting efficiency and prolonged retention time in osteoporotic models, thereby improving bone mineral density and trabecular architecture (Rawat et al., 2016).

Another promising approach involves the use of metallic nanoparticles, such as gold and silver nanoparticles, which exhibit strong affinity for bone minerals. These nanoparticles can be modified with osteotropic molecules to selectively target osteoclasts and osteoblasts, enhancing the therapeutic effects of natural compounds while minimizing systemic toxicity (Gao et al., 2023). Furthermore, mesoporous silica nanoparticles (MSNs) have emerged as versatile drug carriers due to their large surface area, tunable pore size, and controlled release properties. MSNs loaded with natural polyphenols have shown enhanced osteogenic differentiation and bone regeneration capabilities (Pinna et al., 2021; Long et al., 2024).

#### 6.6.2 Liposomal and exosome-based drug delivery

Liposomal formulations have been widely investigated for improving the bioavailability and stability of hydrophobic natural compounds. Liposomes, composed of phospholipid bilayers, protect bioactive molecules from enzymatic degradation and facilitate their controlled release at the target site. Several studies have demonstrated that liposomal encapsulation of flavonoids such as quercetin and kaempferol enhances their osteoprotective effects by promoting osteoblast differentiation and inhibiting osteoclast-mediated bone resorption (Sheng et al., 2024; Zhang et al., 2025). Additionally, liposomal curcumin formulations have exhibited superior anti-inflammatory and antioxidant effects in osteoporosis models, contributing to enhanced bone regeneration (Partoazar and Goudarzi, 2022; Yeh et al., 2015). Exosomes, naturally occurring extracellular vesicles, have also gained attention as potential drug delivery vehicles for bone-targeted therapy. These nanovesicles can be engineered to carry osteogenic phytochemicals, improving their stability and targeted delivery. Recent studies have shown that exosome-based delivery of yam-derived exosome-like nanovesicles enhances bone formation while reducing osteoclast activity, making it a promising strategy for osteoporosis treatment (Hwang et al., 2023; Zhao et al., 2024).

#### 6.6.3 Bisphosphonate-functionalized drug conjugates

Bisphosphonates (BPs), a class of anti-resorptive agents widely used for osteoporosis management, exhibit strong affinity for hydroxyapatite, allowing targeted drug delivery to bone tissues. Conjugating natural osteoprotective compounds with bisphosphonates enhances their skeletal accumulation while reducing off-target distribution. Studies have demonstrated that bisphosphonate conjugates improve the osteoprotective efficacy of flavonoids, reducing bone loss in osteoporotic models (Mbese and Aderibigbe, 2021). Additionally, bisphosphonate-functionalized nanoparticles have been designed to enhance drug retention at resorption sites. For instance, loaded alendronate-conjugated nanoparticles have been shown to selectively accumulate in bone, leading to sustained drug release and prolonged therapeutic effects. This approach minimizes systemic toxicity while maximizing the bone-protective effects of natural compounds (Jing et al., 2021).

#### 6.6.4 Smart hydrogels and bone scaffolds

Hydrogels have gained prominence as drug carriers for localized and sustained release of osteoprotective agents. These three-dimensional polymeric networks can be engineered to release drugs in response to specific physiological triggers, enhancing therapeutic precision. Injectable hydrogels loaded with polyphenols have shown improved bone regeneration by maintaining a controlled drug release profile over extended periods. Moreover, hydrogels functionalized with bioactive peptides can promote osteoblast differentiation and enhance bone matrix formation (Xv et al., 2025; Wu et al., 2025). Scaffold-based drug delivery systems have also emerged as an effective approach for bone tissue engineering. Biodegradable scaffolds composed of collagen, chitosan, or calcium phosphate have been utilized for sustained delivery of natural compounds, facilitating bone regeneration while providing structural support. In particular, bioactive scaffolds loaded with quercetin or berberine have demonstrated significant potential in promoting osteogenesis and inhibiting osteoclast-mediated bone resorption (Gong et al., 2022).

#### 6.6.5 Micelle-based and prodrug strategies

Micelle-based delivery systems offer a promising solution for improving the solubility and cellular uptake of hydrophobic natural compounds. These self-assembling amphiphilic molecules enhance drug stability, prevent premature degradation, and facilitate targeted delivery. Recent studies have shown that micelle-encapsulated flavonoids exhibit enhanced osteogenic potential, leading to increased bone formation in osteoporosis models (Zhang et al., 2023). In addition to micelles, prodrug strategies have been employed to enhance the bioavailability of natural compounds. Prodrugs remain inactive until they are enzymatically converted into active molecules at the site of bone resorption. This approach minimizes systemic side effects while ensuring efficient bone-targeting. For example, enzyme-responsive prodrugs of myricetin have demonstrated superior pharmacokinetic profiles, improving bone density and strength (Xi et al., 2022).

Targeted drug delivery systems present a revolutionary approach to overcoming the limitations of natural compounds in osteoporosis treatment. By leveraging nanotechnology, liposomal encapsulation, bisphosphonate conjugation, hydrogel-based scaffolds, micelle-based delivery, and prodrug activation, these strategies enhance the therapeutic potential of bioactive molecules while minimizing systemic side effects. The combination of natural osteoprotective agents with advanced delivery systems offers a promising avenue for osteoporosis management, paving the way for future clinical applications. Further research should focus on optimizing these delivery mechanisms for human use, ensuring the development of safe, effective, and targeted therapies for osteoporosis.

## 7 Conclusion and perspective

This review underscores the potential of natural compounds targeting TRAFs for osteoporosis treatment. Key natural products, such as dioscin, puerarin, cytisine, shikimic acid, and guaiacol, demonstrate distinct and complementary mechanisms in modulating TRAF-mediated pathways involved in bone remodeling. These compounds address the root of osteoporosis—imbalanced bone turnover by inhibiting osteoclastogenesis and promoting osteoblast differentiation.

Each natural compound exhibits a unique interaction with TRAF-associated pathways. For example, Sparganii Rhizoma and puerarin primarily reduce osteoclast differentiation and inflammatory signaling via TRAF6/NF-κB and MAPK pathways while stimulating osteoblast activity through BMP-2/SMAD pathways. Cytisine’s modulation of the PI3K/AKT pathway and polyphyllin VII’s regulation of oxidative stress through the TRAF6-cSrc-PI3K pathway also highlight their roles in protecting bone from excessive resorption. On the other hand, icaritin and LIQ are the other natural substances that abrogate osteoclast differentiation and activity by attenuating ROS levels and subsequently suppressing ROS-related MAPK and NF-κB signaling pathways. Kacip Fatimah, AR281, further supports these effects by downregulating inflammatory cytokines and enhancing osteoclast apoptosis. These findings suggest that combining these compounds could produce a synergistic effect, effectively addressing both osteoclastogenesis inhibition and osteoblastogenesis stimulation (Figure 3).

Future research should focus on these herbal compounds’ pharmacokinetics, optimal dosing, and safety profiles to pave the way for clinical applications. A significant challenge in the clinical application of herbal compounds is optimizing dosage and improving bioavailability. Many natural compounds are limited by their poor solubility and rapid metabolism, which can reduce their therapeutic efficacy when administered in conventional forms. Recent advancements in drug delivery systems, such as nanoparticle encapsulation, liposomes, and hydrogel formulations, offer solutions to these issues by protecting the compounds from rapid degradation and enhancing their targeted delivery to bone tissues. For instance, nanoparticles like hydroxyapatite could serve as carriers for these compounds, ensuring they are directed precisely to sites of bone remodeling. Future research should focus on developing and testing such delivery mechanisms to improve TRAF-targeting herbal compounds’ stability, absorption, and bioavailability. Given that TRAF-mediated pathways are complex and involve multiple interdependent signaling cascades, a multitarget approach may be more effective than single-agent treatments. Combining compounds such as Sparganii Rhizoma, which targets inflammatory signaling, with polyphyllin VII, known for its antioxidant properties, could produce a more comprehensive therapeutic effect, addressing both inflammation and oxidative stress, two major contributors to osteoclast activation in osteoporosis. Preclinical and clinical studies are necessary to identify synergistic combinations, evaluate their interactions within TRAF pathways, and assess their safety and efficacy in humans. Combining these natural compounds with current osteoporosis drugs, such as bisphosphonates or RANKL inhibitors, could further enhance their therapeutic potential.

The heterogeneous nature of osteoporosis, which can be influenced by genetic, hormonal, and lifestyle factors, presents a strong case for the development of biomarker-driven, personalized therapy. TRAF-related biomarkers could help identify patients who are more likely to benefit from TRAF-targeting therapies, allowing for more precise and individualized treatment. Future research should focus on identifying specific biomarkers associated with TRAF activation and dysfunction in osteoporosis. By assessing TRAF pathway activity in patients, it may become possible to tailor natural compound therapies to individuals based on their unique biochemical profiles, maximizing therapeutic benefit and minimizing potential adverse effects. To bring TRAF-targeting natural compounds into widespread clinical use, rigorous clinical trials are essential to establish their safety, efficacy, and optimal therapeutic protocols. These trials will need to consider dose–response relationships, potential side effects, and long-term outcomes in diverse populations, including postmenopausal women and elderly individuals who are at higher risk for osteoporosis. Regulatory pathways for herbal medicines are often distinct from those for conventional drugs, so additional efforts may be required to meet regulatory standards and ensure quality control of these natural compounds, especially if they are used in combination therapies. The impact of natural compounds on TRAF signaling pathways may extend beyond the prevention of bone resorption. Given that certain compounds also promote osteoblast differentiation, TRAF-targeting natural products hold potential for osteoporosis treatment and bone regenerative therapies. This could have implications for other bone-related disorders, such as osteoarthritis and bone healing after fractures. Further exploration of these broader applications, alongside advances in tissue engineering and regenerative medicine, could establish new therapeutic uses for these compounds in orthopedic and dental medicine.

In summary, natural compounds targeting TRAF pathways offer an innovative, multipronged approach to osteoporosis treatment. With continued research in optimizing their efficacy, safety, and delivery, as well as clinical validation, these compounds could reshape the management of osteoporosis and serve as a model for integrating natural products in chronic disease management. Future research and development in these areas will be crucial to realizing the full potential of TRAF-targeting natural compounds, providing a new paradigm in both osteoporosis prevention and bone health preservation.
