# Supplementary material for: TNF receptor-associated factors: promising targets of natural products for the treatment of osteoporosis
Source: Front Physiol. 2025 May 27;16:1527814. doi: 10.3389/fphys.2025.1527814 (PMC12148923; doi:10.3389/fphys.2025.1527814)
Supplement: Supplementary file 1 [file Table1.docx]

**Table 1.** Therapeutic natural products targeting RANKL/RANK/TRAFs in osteoporosis

| **Natural product name** | **Source** | **Model** | **Dose** | **Other targets** | **Signaling pathway** | **Effects** | **Ref** |
| --- | --- | --- | --- | --- | --- | --- | --- |
| Nodakenin | Radix Angelicae biseratae | In vivo and in Vitro | 50-200 μM | Runx2, col-I and SP7 | PI3K/Akt/mTOR,  Akt/GSK3β, and NF-κB | “NK significantly promoted osteogenic differentiation of BMSCs through the PI3K/Akt/mTOR pathway while inhibiting osteoclastogenesis via Akt/GSK3β and NF-κB signaling pathways in vitro.  Oral administration of NK effectively attenuated bone loss in OVX mice, improving bone mass and microarchitecture without causing liver or kidney toxicity.  NK demonstrated no significant cytotoxicity at effective doses, supporting its potential as a safe and effective treatment for osteoporosis.” | [1] |
| Morroniside | Cornus officinalis Sieb | In vivo and in vitro |  | ALP, OCN, and Runx2, | NF-κB/MAPK signaling pathway | “Morroniside promotes osteogenic differentiation of BMSCs and enhances mineralization and bone formation.  Morroniside improves bone microstructure and density while enhancing biomechanical properties in mouse models of inflammatory bone loss.” | [2] |
| Catalpol | Rehmanniae Radix | In vitro | 0, 1, 2, 3 or 5 mg/ml | miR-124-3p, DNMT3b | miR-124-3p/DNMT3b/TRAF6 | “Catapol has potential in alleviating bone loss and facilitating osteoblast differentiation, with its detailed mechanism in osteoblast differentiation needing further exploration.” | [3] |
| Methanolic extract of O.umbellata (MOU) | Oldenlandia umbellata | In vitro | 500 μg/mL | TRAF6, NFATc1, c-Jun, c-Fos, CTSK, and β-catenin | ↓wnt/β-catenin/↓Runx2  ↓TRAF6/↓NF↓ATc1/C-fos/↓C-jun/↓CTSK | “MOU could enhance the proliferation of osteoblasts by inhibiting GSK3β in the wnt/β-catenin/Runx2 signalling and inhibit RANKL stimulated osteoclast formation via inhibiting TRAF6/NFATc1/C-fos/C-jun/CTSK in the RANK-RANKL signaling.” | [4] |
| Marantodes pumilum | Leaf aqueous | In vivo and in vitro | 50 and 100 mg/kg/day | ↑OPG, Wnt3a, ↑LRP-5, ↑Frizzled, Dvl, ↑β-catenin, RUNX, and ↑Bmp-2  ↓RANKL, ↓Traf6, and ↓NF-kB | RANK/↓RANKL/↑OPG | “MPLA helps to protect against bone deterioration in estrogen deficiency state, mitigate deterioration in cancellous bone microarchitecture and the loss of bone glycogen and collagen content by decreasing Levels of RANKL, Traf6, and NF-kB but not RANK in bone and stimulating OPG, Wnt3a, LRP-5, Frizzled, Dvl, β-catenin, RUNX, and Bmp-2.” | [5] |
| Icariside I (GH01) | Epimedium | In vivo and in vitro | 5 and 50 mg/kg body weight | ↓NFATc1, ↓c-FOS, and ↓TRAP | ↓RANKL/ ↓TRAF6/MAPK-p38/NFATc1 | “GH01 dose-dependently promoted osteoblast differentiation and formation through inhibition of adipogenesis and acceleration of energy metabolism of osteoblasts.” | [6] |
| Resveratrol |  | In vitro | 5, 10, and 20 mol/L | ↓Calcitonin receptor, ↓NFATC1, ↓MMP-9, ↓cathepsin K (CTSK), and ↓TRAP, ↓p-IKB-α and ↓NF-kB65   ↓TNF-α, ↓IL-1β, and ↓IL-6 | ↓RANKL/↓RANK/↓TRAF6  ↑miR-181a-5p/↓TRAF6/↓TAK1 | “Res can reduce the RAW 264.7 cell differentiation into osteoclasts and relieve LPS-stimulated osteoporosis, and the underlying mechanism may be associated with the Res-inhibited activity of the TRAF6/TAK1 pathway through the increased miR-181a-5p expression.” | [7] |
| curcumenol | Curcuma zedoaria | In vivo and in vitro | 50, or 100 μM | ↓inositol polyphosphate multikinase (IPMK), ↓TRAF6, ↓NFATc1, ↓CTSK, and ↓ATP6v0d2 | ↓RANKL/↓RANK/↓TRAF6/↓MAPK/↓NF-κB | “CUL could impair the stability of TRAF6 enhanced by IPMK and suppress excessive osteoclast activity in estrogen-deficient mice to treat osteoporosis.” | [8] |
| Liquiritin (LIQ) | An flavanone derivative | In vivo and in vitro | 0.01, 0.05, and 0.1 mM | ↓Acp5, ↓Cathepsin K, ↓Atp6v0d2, ↓Nfatc1, c-Fos, and ↓MMP-9 | ↓RANKL/↓RANK/↓TRAF6/↓MAPK/↓NF-κB | “LIQ impedes osteoclast formation triggered by RANKL and the subsequent reduction in bone mass by mitigating ROS levels and suppressing the Ca^2+^/MAPK-NFATc1 signaling pathway.” | [9] |
| Xanthohumol (XN) | prenylflavonoid | In vivo and in vitro | 5 μM | ↓CtsK, ↓NFATc1, ↓Trap, and ↓Ctr | RANKL/TRAF6/ ↓NF-κB | “ XN can inhibit osteoclastogenesis in vitro and in vivo through suppressing RANK/TRAF6 interaction, and blocking NF-κB and Ca^2+^/NFATc1 signaling pathway.” | [10] |
| Icaritin (ICT) | prenylflavonoid | In vivo and in vitro | 100 nM | ↓TRAF6, ↓c-Fos, ↓NFATc1 | RANKL/TRAF6/ ↓NF-κB, ↓MAPK/AP-1  ↓TRAF6/↓c-Src/↓PI3K | “ICT, by downregulating TRAF6, coordinates inhibition of NF-κB, MAPK/AP-1, and ROS signaling pathways to reduce expression and activity of NFATc1.” | [11] |
| curcumin |  | In vivo and in vitro | 5, 10 or 15 μM | ↑Atg5, ↑Atg7 or ↑Beclin1,↑LC3, ↑E64d and PEPS A | ↓RANKL/↓RANK/↓TRAF3 | “Curcumin can directly enhance the autophagic activity of osteoclast precursors (OCPs), which inhibits its anti-osteoclastogeneic effects.” | [12] |
| Trilobatin (Tri ) | Flavonoid glycoside |  | 5-20 μM | ↓MMP-9 and ↓cathepsin K, ↓CA II, ↓integrin β3, ↓TRAF6, p-Pyk2, ↓c-Cbl, and c-Src | ↓RANKL/↓RANK/↓TRAF6-c-Src-Pyk2-c-Cbl | “Tri exerts significant anti-osteoporotic activity by dampening RANKL-induced osteoclastogenesis and bone resorption through downregulation of TRAF6-c-Src-Pyk2-c-Cbl signalling pathway and significantly antagonized bone loss in estrogen-deficient OVX mice by increasing serum OPG/RANKL ratios and BMD of both femur and tibia bones.” | [13] |
| Icariin | Flavonoid glycoside | In vitro | 10 µM | ↓c-Fos, ↓RANK, ↓TRAP, and ↓Atp6v0d2 | RANKL/TRAF6-TAK1/ ↓NF-κB/↓ERK | “Icariin suppressed RANKL-induced osteoclast differentiation via downregulation of TRAF6, inhibition of ERK1/2 and NF-κB activation, and subsequently decreased expression of c-Fos, RANK, TRAP, Atp6v0d2, and NFATc1..” | [14] |
| RANK-tet |  | In vitro | 1 μg/ml |  | ↓RANK/↓TRAF6 | “the unique binding mode of RANK-tet to TRAF-C through multiple residues including Arg392, Phe410, Lys469, Phe471, and Tyr473, would explain why RANK-tet efficiently inhibits the interaction between RANK and TRAF6 to inhibit osteoclastogenesis.” | [15] |
| NAE inhibitor MLN4924 |  | In vivo and in vitro | 10 mg/kg |  | RANKL/ TRAF6/TAK1/NFATc1 | “MLN4924 significantly reduced osteoporosis in an ovariectomy- and sRANKL-induced osteoporosis mouse model in vivo and reversed NAE-mediated neddylation, thereby inhibiting RANKL-activated TRAF6–TAK1–NFATc1 signaling during osteoclast differentiation and osteoporosis.” | [16] |
| Trimethylamine-N-oxide (TMAO) | Gut microbiota (GM)-derived metabolite | In vivo and in vitro | 100–400 μM | ↑Calcr, ↑Traf6, ↑Dcstamp, ↑Acp5, ↑C-Fos, and ↑NFATc1 | ROS-dependent NF-κB | “TMAO could promote osteoclast differentiation and induce bone loss in mice by activating the ROS-dependent NF-κB signaling pathway.” | [17] |
| AR281 | Lactobacillus brevis | In vivo and in vitro |  | ↓IL-1, ↓IL-6, and ↓IL-17, ↓TNFα, ↓RORα, and ↓RORγ  ↑claudin-2, ↑claudin-3, ↑ZO-1, and ↑occludin | ↓RANKL/↓RANK/↓TRAF6/NF-κB | “AR281 suppressed the RANKL/OPG ratio by downregulating the expression of pro-inflammatory osteoclastogenic cytokines, thereby inhibiting the binding of RANKL and RANK on OCPs. AR281 further attenuated the osteoclast differentiation by modulating the TRAF6/NF-κB/NFATc1 pathway after the binding of RANKL and RANK.” | [18] |
| LC38‐CS | Lactobacillus curvatus Wikim 38 | In vitro |  | ↓PRMT1, ↓F‐actin and ↓ADMA | ↓RANKL/↓RANK/↓TRAF6/ERK/↓NF-κB | “LC38‐CS inhibited RANKL‐induced osteoclast differentiation by the downregulation of molecular mechanisms and exerted anti‐osteoporotic effects.” | [19] |
| Pterostilbene isothiocyanate (PTER-ITC) |  | In vivo and in vitro | 5 and 10 µM |  | RANK/↓TRAF6 | “PTER-ITC not only inhibited RANK/TRAF6 interaction in osteoclastogenic cells, but also suppressed the expression of osteogenic markers in osteoblasts.” | [20] |
| 6-acetylacteoside (6-AA) | Cistanche deserticola | In vivo and in vitro | 10, 20, and 40 mg/kg/day | ↓NF-κB, ↓NFATc1, ↓RANK, ↓RANKL and ↓TRAF6  ↑OPG, ↑PI3K and ↑AKT | ↓RANKL/↓RANK/↑OPG | “6-AA could prevent bone loss by regulating the OPG/RANK/RANKL signaling pathway, including NF-κB pathway inactivation and PI3K/AKT signaling activation, which ultimately inhibited osteoclastogenesis and promoted bone formation.” | [21] |
| ADWGGPLPH | Wheat germ peptide | In vivo and in vitro |  |  | OPG/↓RANKL/↓RANK/↓TRAF6 | “ADWGGPLPH could improve osteoblasts' proliferation and differentiation activity and effectively inhibit osteoclasts’ differentiation by regulating the OPG/RANKL/RANK/TRAF6 pathway.” | [22] |
| circBBS9 |  | In vivo and in vitro | 1 μg of siRNA or shRNA per well | ↓NFATc1, ↓c-FOS**,** ↓integrin-β3, ↓CTSK, and ↓V-ATPase-d2 | ↓miR-423-3p/↓Traf6 | “knockdown of circBBS9 in pOCs inhibits OC multinucleation through the circBBS9/miR-423-3p/Traf6 axis, indicating that circBBS9 is an effective posttranscriptional regulator.” | [23] |
| Mogrol | Siraitia grosvenorii | In vivo and in vitro | 20 μM | ↓CTSK, ↓ACP5, ↓MMP9 | ↓RANKL/↓RANK/↓TRAF6/NF-κB and MAPK | “Mogrol suppresses osteoclastogenesis and resorption by inhibiting the RANKL-dependent TRAF6/NF-κB/MAPK signaling pathway and relieved bone mass loss in postmenopausal mice.” | [24] |
| Gadus morhua eggs (Gds) |  | In vitro |  |  | OPG/RANKL/TRAF6 | “Gds could reduce bone resorption, increase bone formation, suppress high bone turnover, and improve osteoporosis in ovariectomized rats via OPG/RANKL signal axis, closely related to the serum metabolism.” | [25] |
| Puerarin | Puerariae lobate | In vivo and in vitro | 500-1000 ​μM | ↓TRAP-5b, ↓CTx1 | ↓integrin-β3-Pyk2/↓Cbl/↓Src | “Puerarin prevented the bone loss in OVX rat through suppression of osteoclast activation and bone resorption, by inhibiting integrin-β3-Pyk2/Cbl/Src signaling pathway, without affecting osteoclasts formation or apoptosis.” | [26] |
| Sparganii Rhizoma (SR) | Sparganium stoloniferum | In vivo and in vitro | 125, 250, 500, 1,000 μg/ml | ↑RUNX2, Osterix, and p-SMAD1/5 | ↓TRAF6/↓MAPK/NF-κB/↓NFATc1/↓c-Fos  ↑BMP-2/↑SMAD | “SR inhibited osteoclast differentiation, function, and bone resorption through the TRAF6/MAPK/NF-κB/NFATc1/c-Fos pathways and stimulates osteoblast differentiation by increased protein expression of the BMP-2/samd signaling pathway.” | [27] |
| Roburic acid (RA) | Radix Gentianae Macrophyllae | In vitro | 1 to 10 μM | NFATc1, antioxidative response element (ARE), and heme oxygenase (HO)-1 | ↓RANKL/↓RANK/↓TRAF6/ERK/↓NF-κB | “RA demonstrated the ability to inhibit osteoclast differentiation in bone marrow macrophages without toxicity, while also showing no adverse effects on osteoblast formation. Mechanistically, RA reduced osteoclast activity by suppressing key signaling pathways, including NF-κB, ERK phosphorylation, and calcium oscillations, and enhanced antioxidative responses.” | [28] |
| SPIO@HA | Hydroxyapatite coated superparamagnetic iron oxide nanoparticles | In vivo and in vitro | 10 mg Fe/kg | ↑OPG, ↑CSF2, ↑CCL2 | ↓TRAF6−↑p62−↑CYLD   TGF-β/PI3K/AKT | “SPIO@15HA showed inhibitory effect on formation of RANKL-induced TRAP-positive multinucleate cells and actin rings during osteoclastogenesis.” | [29] |
| Arsenic (As) |  | In vitro |  | ↓NFATc1 | RANKL/TRAF6-TAK1/ ↓NF-κB | “ The expression of TRAF-6 and NF-κB pathway was affected by fluoride and As co-exposure in osteogenic differentiation, and As could antagonize the promoting effect of F on the expression of TRAF-6, TRAP, RANKL, RANK, NF-κB, and NFAT-c1 in these exposure levels.” | [30] |
| Iron oxide nanoparticles (IONPs) |  | In vivo and in vitro | 0.1 mg Fe/mL | ↓Calcr, ↓Ctsk, and ↓c-Src | ↑p62/↓CYLD/↓TRAF6  RANKL/TRAF6-TAK1/↓MAPK, ↓NF-κB | “he IONPs could upregulate p62 expression to enhance the binding of CYLD to the TRAF6-p62 -CYLD complex, leading to [deubiquitination](https://www.sciencedirect.com/topics/medicine-and-dentistry/deubiquitination) of TRAF6 and blockage of RANKL induced sequential signal transduction such as NF-κB and MAPK signals.” | [31] |
| Oxygen ultra-fine bubbles (OUB) |  | In vivo and in vitro | 5 mg | ↓TRAF6, ↓c-Fos, ↓NFATc1, ↓TRAP, ↓cathepsin K, ↓DC-STAMP | RANKL/TRAF6-TAK1/↓MAPK, ↓NF-κB | “OUB significantly inhibited osteoclastogenesis by inhibiting RANK-TRAF6-c-Fos-NFATc1 signaling, RANK-p38 MAPK signaling, and TRAP/Cathepsin K/DC-STAMP mRNA expression in a concentration-dependent manner.” | [32] |
| Kacip Fatimah | M. pumilum leave aqueous extract | In vitro | 50 and 100 mg/kg/day | ↓RANKL, ↓NF-κB p65, ↓IKKβ, ↓IL-6, ↓IL-1β and Keap-1  ↑RANK, ↑OPG, ↑BMP-2, Type-1 collagen, ↑Runx2, ↑TRAF6, ↑Nrf2, ↑NQO-1, HO-1, ↑SOD and CAT | ↓RANKL/↑RANK/↑OPG | “MPLA in particular at high dose has been found able to overcome the adverse effect of sex-steroid deficiency and DM on the bone via multiple pathways that ultimately results in the stimulation of bone proliferative activity which leads to increase in bone mass and its density.” | [33] |
| Forsythoside-β |  | In vivo and in vitro | 50 μM | ↓NFATc1, ↓Ctsk, ↓Dc-stamp, Ctr, ↓MMP‐9, ↓ACP5, and ↓NF-KB | ↑Nrf2/↓mir-214-3p/↑Traf3 | “FB, a traditional PHG identified as Nrf2 activator, was capable to inhibit osteoclast differentiation in vitro and prevent bone resorption in vivo.” | [34] |
| Gamabufotalin (CS-6) | Chansu | In vivo and in vitro | 5, 50, 100, and 150 nM | TRAP, CTSK, DC-STAMP, MMP9, and β3 integrin | ↓RANKL/↓RANK/↓TRAF6/ERK/↓NF-κB | “CS-6 can suppress RANKL/TRAF6-induced early activation of NF-κB and ERK/MAPK signaling pathways, which consequently suppressed the transcription activity of c-Fos and NFATc1.” | [35] |
| Rosavin |  | In vivo and in vitro | 1.25, 2.5, or 5 µM | ↓TRAP, ↓cathepsin K, ↓MMP-9, CTR, ↓TRAF-6, RANK, ↓NAFTc1, and c-fms  Runx2, | ↓RANKL/↓RANK/↓TRAF6/↓MAPK/↓NF-κB | “Rosavin promoted osteogenesis and suppressed RANKL-induced osteoclastogenesis and inhibits osteoclastogenesis at an early stage by suppressing the NF-κB and MAPK signaling pathways.” | [8] |
| Norlichexanthone (NOR) | Plant endophyte | In vivo and in vitro | 1 mg/kg |  | ↓RANKL/↓RANK/↓TRAF6 | “NOR inhibited RANKL-induced association of ERα and TRAF6 to prevent ERα-mediated TRAF6 activation via Lys63-linked ubiquitination and exhibited potent anti-osteoporosis efficacy in an ovariectomized mouse model.” | [36] |
| Puerarin | Pueraria radix | In vivo and in vitro | 0, 10, or 100 μM | ↓NFATc1, ↓MMP9, ↓CTSK, Acp5 and c-Fos  TRAF6 and NADPH oxidase 1 (NOX1) and HO-1 | ↓TRAF6/↓ROS-dependent ↓MAPK/↓NF-κB | “puerarin inhibits in vivo and in vitro osteoclast differentiation and activity by decreasing ROS levels and subsequent inhibition of ROS-induced MAPK and NF-κB signaling pathways.” | [37] |
| 2’-Acetylacteoside-(2’-AA) | Cistanche deserticola | In vivo and in vitro | 10 and 20mg/kg/day | RANK, ↓TRAF6, IκB kinase β, ↓NF-κB and ↓NFATc1 | ↓RANKL/↓RANK/↓TRAF6/↓NF-κB/NFATc1 | “2’-AA could prevent bone loss on OVX mice through RANKL/RANK/TRAF6 mediated NF-κB/NFATc1 pathway, which evidenced by the expression levels of TRAF6, RANK, IKKβ, NF-κB, NFATc1 were down-regulated by 2’-AA treatment as compared to control group.” | [38] |
| schisandrin A (Sch) | Schisandra chinensis | In vivo and in vitro | 200 μmol/L | ↓CTSK, ↓MMP9 and ↓V‐ATPase | ↑Nrf2/↓RANKL/↓RANK/↓TRAF6/↓NF-κB | “ Sch enhanced the expression of Nrf2 by regulating the degradation of Nrf2 and suppressed phosphorylation of P65 and its nuclear translocation, as well as the degradation of IκBα thereby protecting against OVX‐induced bone loss by suppressing ROS via Nrf2.” | [39] |
| Cudratrixanthone U (CTU) | Maclura tricuspidata Bureau | In vitro | 0.5, 1, 2, and 5 μM | MMP-9, DC-STAMP, CTSK, CCL4 | RANKL/TRAF6-TAK1/↓MAPK/↓NF-κB | “RANKL increased TRAF6 and TAK1 association, which was inhibited by CTU. These results suggest that CTU targets TRAF6-TAK1 complex formation to inhibit osteoclast differentiation.” | [40] |
| Antarctic krill oil (AKO) and arachidonic acid-rich oil (AAO) |  | In vitro | 200 mg kg^−1^ | c-fos, NFATc1, TRACP, MMP-9, and Cath-K | OPG/↓RANKL/↓TRAF6/↓NF-κB | “AKO could significantly improve osteoporosis via the OPG/RANKL/NF-κB pathway mediated by PGE_2_/EP_4_ to inhibit osteoclastogenesis, whereas AAO aggravated osteoporosis via the same pathway.” | [41] |
| Cytisine | Leguminosae (Fabaceae) family | In vivo and in vitro | 200 μmol/L | ↓NFATc1, ↓Cathepsin K, ↓MMP‐9 and ↓TRAP | ↓RANK/↓RANKL/ ↓JNK/ERK/p38‐MAPK, ↓IκBα/p65‐NF‐κB  ↓PI3K/↓AKT/↓NFATc1 | "Cytisine can also suppress the action of RANK and TRAF6 at absence of RANKL, implying that Cytisine maybe has an effect for RANKL‐induced recruitment of TRAF6 by RANK at endogenous cells." | [42] |
| Cinchonine |  | In vitro | 10 mg/kg | ↓NFATc1, ↓PGC1β, IkBα, JNK1, p38, c‐Fos, and CTSK | ↓TRAF6/ ↓TAK1 and AKT | “CN may inhibit RANKL‐induced osteoclast differentiation by regulating TAK1 and AKT through targeting TRAF6. CN also inhibits the maturation and resorption function of osteoclasts and promotes osteogenesis.” | [43] |
| Ortho-silicic acid |  | In vivo and in vitro | 10 20 μM | ↓NFATcl, ↓TRAF6, ↓NF-κB P50, ↓NF-κB P52, RANK, ↓integrin β3, ↓CTSK, ↓DC-STAMP, and TRAP | OPG/↓RANKL/↓TRAF6/↓NF-κB | “OSA could also suppress osteoclast differentiation through the RANKL/RANK/OPG signaling pathway, which regulates bone resorption.” | [44] |
| Neobavaisoflavone (NBIF) | Psoralea corylifolia | In vivo and in vitro | 30 mg/kg | ↓NFATc1, ↓P50, ↓P65, IκB, ↓F‐actin | ↓RANKL/↓RANK/↓TRAF6/↓NF-κB, ERK, JNK, P38 | “NBIF suppressed RANKL‐induced NF‐κB, MAPKs pathways activation and abrogated the interaction of RANK and TRAF6 during osteoclastogenesis.” | [45] |
| Salvia miltiorrhiza ethanol extract (SME) | Salvia miltiorrhiza | In vivo and in vitro | 50–200 mg/kg | ↓RANKL, ↓osteocalcin, BALP, ↓TRAF6 and ↓NFATc1 | OPG/↓RANKL/↓RANK/↓TRAF6 | “SME suppressed the loss of trabecular bone via suppressing bone resorption and osteoclast differentiation both in OVX and NM mice.” | [46] |
| Shikonin | shikonin tinctoria | In vivo and in vitro | 1.8 μM | ↓NF-κB (IκB), ↓P50, ↓P65, ERK, ↓JNK, and ↓P38, MMP-9, cathepsin K, TRAP, and CTR | ↓RANKL/↓RANK/↓TRAF6/↓NF-κB, ERK, JNK, P38 | “Shikonin reduced osteoclastogenesis in vivo and in vitro. The inhibitory effects were induced by suppressing the interaction between RANK and TRAF6 and the downstream MAPK and NF-κB signaling pathways.” | [47] |
| Muscone |  | In vivo and in vitro | 10, 20, and 40 μM | NFATc1, CTR, TRAP, cathepsin K, and MMP-9  TNF-a and IL-6 | RANKL/TRAF6-TAK1/↓MAPK/↓NF-κB | “Muscone inhibited the binding of TRAF6 to RANK induced by RANKL, thus blocking NF-kB and MAPK pathways, and down-regulating related gene expression; finally inhibited osteoclastogenesis and osteoclast function by blocking RANK-TRAF6 binding, as well as downstream signaling pathways in vitro.” | [47] |
| Guaiacol | Angelica sinensis | In vivo and in vitro | 0.25, 0.5 and 1.0 μmol/L | ↓p65, ↓p50, IκB, ↓ERK, ↓JNK, ↓c‐fos, ↓p38, and Akt  ↓Cathepsin K, ↓CTR, ↓MMP‐9 ↓TRAP, ↓CTX‐1 and ↓TRAcp5B | RANKL/TRAF6-TAK1/↓MAPK, ↓NF-κB, and ↓Akt | “Guaiacol blocked interactions between RANK and TRAF6 and C‐Src, and suppressed osteoclastogenesis by inhibiting the activation of the NF‐κB, MAPK, and AKT pathways and the Ca^2+^ signalling pathway.” | [48] |
| Polyphyllin VII |  | In vitro | 1, 10, and 30 μM |  | RANKL/↓TRAF6/↓cSrc/↓PI3k/ GTP-Rac1 and ↓Nox1 | “Polyphyllin VII inhibited differentiation of BMMs into osteoclasts through suppressing synthesis of intracellular ROS, which was mediated by the signal transduction of the TRAF6–cSrc–PI3k pathway as well as activation of GTP-Rac1 and Nox1.” | [49] |
| C. deserticola extract (CDE) | Cistanche deserticola | In vitro | 200–800 mg/kg/day | ↓NFAT2, and c-Fos | ↓RANKL/↓RANK/↓TRAF6/ ↓NF-κB and ↓PI3K/AKT | “CDE possessed potential anti-osteoporotic activity and this effect was, at least in part, involved in modulation of RANKL/RANK/TRAF6-mediated NF-κB and PI3K/AKT signaling as well as c-Fos and NFAT2 levels.” | [50] |
| l‐tetrahydropalmatine (l‐THP) | corydalis | In vivo and in vitro | 4 mg/kg/d | P50, P65, IκB, NFATc1, ERK, JNK and P38 | RANKL/TRAF6-TAK1/↓MAPK, ↓NF-κB | “l‐THP suppressed osteoclastogenesis by blocking RANK‐TRAF6 interactions and inhibiting NF‐κB and MAPK pathways and significantly inhibited ovariectomy‐induced bone loss and osteoclastogenesis in mice.” | [51] |
| Lingzhi-8 (rLZ-8) | Ganoderma lucidum | In vivo and in vitro | 0.5, 1 and 2.5 μg/ml |  | ↓RANK/↓TRAF6/↓JNK | "rLZ-8 inhibited osteoclastic differentiation and promoted osteoclastic apoptosis. reversed the loss of bone mass in osteoporotic rats with little toxic effects on major organs.” | [52] |
| RTA-408 |  | In vivo and in vitro | 0, 5, 10, and 20 nM | ↓c-Fos and ↓Nfatc1 | ↓RANKL/↓RANK/↓TRAF6/ ↓NF-κB/↓STING–IFN-β | “RTA-408 inhibits NF-κB signaling by suppressing the recruitment of TRAF6 to STING, in addition to attenuating osteoclastogenesis and OVX-induced bone loss in vivo.” | [53] |
| Pristimerin |  | In vitro | 5, 10, 25, and 50 μM | ↓Creatine kinase (CK), ↓Alkaline phosphatase (ALP), ↓TRAP, collagen type I fragments (CTX), ↓bone Gla-protein (BGP), and ↓osteocalcin (OC) | RANKL/TRAF6-TAK1/ ↓NF-κB  ↑PI3k/↑Akt | “Pristimerin effectively treats osteoporosis by reducing stimulation of the RANKL signalling pathway and restoring normal levels of TRAF-6 and NF-κB, thereby reducing osteoclastic activity in bone tissue.” | [54] |
| Extracts from plastrum testudinis (PTE) | Plastrum testudinis | In vivo and in vitro |  | ↑STE20, ↑IGF1R, and ↑p38 ↑MAPK  /↓TRAF6 |  | “PTE promotes the proliferation and osteogenic differentiation of rBMSCs by upregulating p38 MAPK, STE20, and IGF1R and downregulating TRAF6 expression.” | [55] |
| Shikimic acid | Illicium verum Hook.f | In vivo and in vitro | 21.25, 42.5, and 85 μg/mL | NFATc1, TNF-α, C-telopeptide of type I collagen (CTX-1), TRAcp 5B, and IL-6 | RANKL/TRAF6-TAK1/↓MAPK, ↓NF-κB | “Shikimic acid inhibited osteoclastogenesis and osteoclast function by blocking RANK ligand-induced recruitment of TRAF6, as well as downstream signaling pathways in vitro. Shikimic acid also reduced ovariectomy-induced osteoclastogenesis and bone loss in vivo.” | [56] |
| Tomatidine | Solanaceae | In vivo and in vitro | 2, 4, and 8 µM | ↓JNK/↓p38, ↓NF-κB, ↓Akt | RANKL/TRAF6-TAK1/↓MAPK, ↓NF-κB | “Tomatidine suppresses osteoclastogenesis and mitigates estrogen deficiency-induced bone mass loss by modulating TRAF6-mediated signaling.” | [57] |
| Sanggenon C (SC) | stem bark of Cortex Mori | In vivo and in vitro | 1 μM, 3 μM and 10 μM | ↓TRAF6*,*↓ NFATc1*,*↓CTSK*,* and TRAP  ↑Runx2 and ↑Collagen I | ↓RANKL/↓RANK/↓TRAF6 | “SC could stimulate osteoblast and inhibit osteoclast formation and function in vitro, which indicated that SC may exhibit anti-osteoporosis activity. Expression of Runx2 and Collagen I were found to be upregulated in the SC-treated group, which suggested SC could promote the differentiation of osteoblasts.” | [58] |
| Daphnetin | Genus Daphne | In vitro |  | ↓TRAF6*,*↓ NFATc1, and ↓c-Fos | RANKL/TRAF6-TAK1/↓NF-κB, Akt/GSK-3β | “Daphnetin prevented the RANKL-induced activation of NF-κB and Akt/GSK-3β pathways in BMMs and exhibited an inhibitory effect on RANKL-induced osteoclastogenesis in vitro.” | [59] |
| Protocatechuic acid (PCA) | Rubus coreanus Miquel, Astragalus membranaceus Bunge, cinnamon, star anise, medicinal rosemary, and Sorghum bicolor L | In vivo and in vitro | 20 mg/kg | TRAF6*,*↓ NFATc1 | ↓RANKL/↓RANK/↓TRAF6 | “the underlying mechanism of PCA in the suppression of bone loss in OVX mice may be associated with the following effects: (1) reduction of serum level of RANKL and increase in OPG; (2) blocking the RANK signaling pathway via downregulation of TRAF6 and NFATc1 expression; and (3) attenuation of cathepsin K and calcitonin receptor expression.” | [60] |
| 18β-glycyrrhetinic acid (18β-GA) | Glycyrrhiza glabra L roots | In vivo and in vitro | 6.9525, 13.905, and 27.81 μg/ml | ↓TRAP, ↓cathepsin K, ↓CTR and ↓MMP-9  p65, p50, and IκB, p65 | RANKL/TRAF6-TAK1/↓MAPK, ↓NF-κB | “18β-GA inhibited RANKL-induced osteoclastogenesis by inhibiting RANK expression in preosteoclasts and blocking the binding of RANK and TRAF6 which lead to the inhibition of NF-κB and MAPK signaling pathways.” | [61] |
| Syringin | Acanthopanax senticosus | In vivo and in vitro | 10, 20 and 40 mg/kg/day |  | RANKL/TRAF6-TAK1/ ↓NF-κB  ↑PI3k/↑Akt | “Syringin prevented bone lost by TRAF6-mediated inhibition of NF-κB and stimulation of PI3K/AKT, and subsequently increasing the OPG/RANKL ratio and inhibiting the osteoclastogenesis, finally promoting bone formation.” | [62] |
| S.miltiorrhiza Bunge (SME) | Salvia miltiorrhiza | In vitro | 50, 100 and 200 mg/kg b.w./day | ↓TRAF6 and ↓NFTAc1, ↓cathepsin K and ↓calcitonin receptor | OPG/↓RANKL/↓RANK/↓TRAF6 | “SML inhibited tibial bone loss, sustained trabecular bone state, and ameliorated bone biochemical markers. In addition, SML administration compared to SEM and LCa reduced serum levels of RANKL, osteocalcin and BALP through increased serum levels of OPG and E_2_ in OVX mice. SML also had more beneficial effects on protection of estrogen-dependent bone loss through blocking expression of TRAF6 and NFTAc1 and produces cathepsin K and calcitonin receptor to develop osteoclast differentiation.” | [63] |
| Protocatechuic acid(PA) |  | In vivo and in vitro | 2, 4, 8, 16 μM | ↓MMP, c-Src, ↓TRAP, ↓TRAF-6, Cathepsin, ↓AP-1 and ↓NFATc1 | ↑Nrf2/RANKL/TRAF6-TAK1/↓MAPK, ↓NF-κB, ↓COX-2 | “PA has potential role in inhibiting osteoclast differentiation through regulation of oxidative stress and inflammation, all of which are primarily mediated through 2 transcription factors NF-kB and Nrf-2. Further, PA induced apoptosis in mature osteoclasts.” | [64] |
| Dioscin |  | In vivo and in vitro | 20, 40 and 60 mg/kg | Cathepsin k,↓AP-1 and ↓NFATc1 | /RANKL/TRAF6-TAK1/↓MAPK, ↓NF-κB, Akt, ↓AP-1 | “Dioscin promoted osteoblastogenesis through up-regulating OPG/RANKL ratio, and inhibited osteoclastogenesis through down-regulating the levels of RANKL induced TRAF6 and the downstream signal molecules including MAPKs, Akt, NF-κB, AP-1, cathepsin K and NFATc1. In addition, dioscin also inhibited TLR4/MyD88 pathway to decrease the levels of TRAF6 and the related proteins.” | [65] |
| PP-AKP | Antarctic krill Euphausia superba | In vitro |  | TRACP, cathepsin K, MMP-9, deoxypyridinoline, C-terminal telopeptide of collagen I (CTX-1), Ca, and P | RANKL/TRAF6/ ↓NF-κB | “PP-AKP can improve osteoporosis by inhibiting bone resorption via suppressing the activation of osteoclastogenesis related NF-κB pathways.” | [66] |
| Total saponin (TS) | Anemone flaccida | In vitro | 0.1-2.5 μg/mL | ↓TRAF6, ↓c-Fos | RANKL/TRAF6-TAK1/↓MAPK, ↓NF-κB | “TS suppresses RANKL-induced osteoclast differentiation and inflammatory bone loss via the down-regulation of TRAF6 level, suppression of JNK and p38 MAPKs and NF-κB activation, and subsequent decreased expression of c-Fos and NFATc1.” | [67] |
| leonurine hydrochloride (LH) |  | In vivo and in vitro | 0.5–2 μM | ↓CtsK, ↓Nfatc1, ↓Trap, IκBα and NF-κB p65 | RANKL/TRAF6-TAK1/ MAPK, ↓NF-κB /AP-1  ↓PI3k/↓Akt | “LH administration attenuated osteoclast activity, thus preventing bone loss caused by estrogen deficiency in mice and suppressed RANKL-induced osteoclastogenesis via RANK-TRAF6, NF-κB, and PI3K/Akt signaling.” | [68] |
| Chloroquine (CQ) |  | In vivo and in vitro | 25 mg/kg i.p, once daily | ↓BECN1, ↓RelB, ↓NFATc1, NIK, p52, and ↓RelA | ↓RANKL/↓RANK/↓TRAF3 | “CQ inhibits OC formation in vitro and in vivo by preventing TRAF3 lysosomal degradation suggests that CQ could inhibit bone resorption in humans by this mechanism.” | [69] |
| Sinomenine (SIN) | Sinomenioum acutum | In vivo and in vitro | 1 mM | ↓p38, ↓JNK  ↓Fra-1, ↓Fra-2, ↓c-↓Fos, ↓c-Src, ↓MMP-9, ↓TRACP | RANKL/↓TRAF6-TAK1/ ↓MAPK↓NF-κB /AP-1 | “SIN suppressed RANKL induced AP-1 and NFAT transcription, as well as the gene expression of NFATc1 and AP-1 components and attenuated osteoclast formation and Mt-induced bone loss by mediating RANKL signaling pathways.” | [70] |
| Phloretin |  | In vitro | ≥10 μM | ↓NFATc1 | RANKL/↓TRAF6/↓NF-κB | “The inhibition of osteoclast differentiation and bone resorption by phloretin entail a disturbance of TRAF6-NFATc1-NF-κB pathway triggered by RANKL.” | [71] |
| Isoliquiritigenin (ISL) | licorice | In vivo and in vitro | 20 μM | ↓NFATc1, ↓NF-κB p65, ↓c-Fos, and β-actin | RANKL/↓TRAF6-TAK1/ ↓MAPK↓NF-κB /AP-1 | “ISL prevented inflammatory bone loss in mice by attenuating osteoclast activity and suppressed RANKL-induced osteoclastogenesis and inflammatory bone loss via RANK–TRAF6, MAPK, IκBα/NF-κB, and AP-1 signaling pathways.” | [72] |
| caffeic acid 3,4-dihydroxy-phenethyl ester (CADPE) | Sarcandra glabra and Teucrium pilosum | In vivo and in vitro | 10 mg/kg | ↓NFATc1, ↓c-Fos | RANKL/↓TRAF6-TAK1/ ↓MAPK↓NF-κB /AP-1 | “CADPE inhibits osteoclastogenesis through inhibition of the MAPK/AP-1 signaling pathway and Ca^2+^ oscillations, which lead to the activation of NFATc1 and the expression of downstream genes. | [73] |
| Silibinin | Silybum marianum | In vivo and in vitro | 1–20 μM | ↓CtsK, ↓MMP-9  ↑Alkaline phosphatase, collagen type 1, ↑connective tissue growth factor, and ↑bone morphogenetic protein-2 | RANKL/↓TRAF6/c-Src | “silibinin markedly attenuated RANK transcription and intracellular adhesion molecule-1 expression elevated by RANKL, thereby suppressing the differentiation of macrophages to multi-nucleated osteoclasts.” | [74] |
| Chaenomelis Fructus (CF) |  | In vivo and in vitro | 1, 10, 100 μg/ml | ↓Cathepsin K, ↓osteoclast-associated immunoglobulin-like receptor (OSCAR), ↓MMP-9, ↓ATPase H+ Transporting V0 Subunit D2 (ATP6v0d2), and ↓carbonic anhydrase II (CA2) | ↓RANKL/↓RANK/↓TRAF6/NFATc1 | “CF inhibited the differentiation of RANKL-induced osteoclasts and their function and effectively ameliorated OVX-induced osteoporosis rats.” | [75] |
| Lycopus lucidus (LL) |  | In vivo and in vitro | 15.2 mg/kg and 152 mg/kg | ↓Carbonic anhydrase II, ↓cathepsin K, ↓osteoclast-associated receptor, and the ↓d2 isoform of the vacuolar ATPase Vo domain | ↓RANKL/↓RANK/↓TRAF/NFATc1 | “LL is able to inhibit osteoclastogenesis and protect bone loss in the OVX-induced osteoporosis model without the influence of hormones like estrogen.” | [76] |
| Yukmijihwang-tang (YJ) |  | In vivo and in vitro | 200 and 400 mg/kg | ↓NFATc1 and ↓c-Fos, ↓TRAP, ↓ATPase, H+ transporting, ↓lysosomal 38 kDa, ↓V0 subunit d2, ↓osteoclast-associated receptor, ↓osteoclast-stimulatory transmembrane protein, ↓dendritic cell-specific transmembrane protein, ↓MMP-9, ↓cathepsin K, and ↓calcitonin receptor | RANKL/↓TRAF6-TAK1/ ↓MAPK↓NF-κB | “YJ diminished RANKL-activated osteoclast differentiation via signaling pathways, including MAPKs, Akt, IκB, NF-κB, and NFATc1, and osteoclast differentiation-related gene expression in vitro. Moreover, YJ restores bone density in OVX-induced bone destruction in vivo.” | [77] |
| Schisandra chinenesis (SC) |  | In vitro | 0.6, 6 and 60 μg/mL | ↓NFATc1, ↓c-Fos, and TNF-α | RANKL/↓TRAF6/↓NF-κB | “SC attenuates RANKL-induced RAW 264.7 cells differentiation into osteoclasts. Furthermore, the effects of SC were associated with the inhibition of TRAF6 recruitment and inactivation of NF-κB pathway, but not MAPKs pathways, leading to the down-regulations of transcription factors including c-fos and NFATc1.” | [78] |

[1] Liu C, Zhao M, Chen J, Xu L, Wang K, Li G. Nodakenin alleviates ovariectomy-induced osteoporosis by modulating osteoblastogenesis and osteoclastogenesis. Eur J Pharmacol 2023;960:176121. https://doi.org/10.1016/j.ejphar.2023.176121.

[2] Xiao J, Han Q, Yu Z, Liu M, Sun J, Wu M, et al. Morroniside Inhibits Inflammatory Bone Loss through the TRAF6-Mediated NF-κB/MAPK Signalling Pathway. Pharmaceuticals (Basel) 2023;16. https://doi.org/10.3390/ph16101438.

[3] Zhang P, Feng Q, Chen W, Bai X. Catalpol antagonizes LPS-mediated inflammation and promotes osteoblast differentiation through the miR-124-3p/DNMT3b/TRAF6 axis. Acta Histochem 2024;126:152118. https://doi.org/10.1016/j.acthis.2023.152118.

[4] Paramasivam S, Perumal SS. Methanolic extract of O.umbellata L. exhibits anti-osteoporotic effect via promoting osteoblast proliferation in MG-63 cells and inhibiting osteoclastogenesis in RANKL-stimulated RAW 264.7 cells. J Ethnopharmacol 2023;315:116641. https://doi.org/https://doi.org/10.1016/j.jep.2023.116641.

[5] Bin Karim K, Giribabu N, Bin Salleh N. Marantodes pumilum (Kacip Fatimah) Aqueous Extract Enhances Osteoblast and Suppresses Osteoclast Activities in Cancellous Bone of a Rat Model of Postmenopause. Appl Biochem Biotechnol 2024;196:821–40. https://doi.org/10.1007/s12010-023-04515-9.

[6] Chen C, Wu M, Lei H, Cao Z, Wu F, Song Y, et al. A Novel Prenylflavonoid Icariside I Ameliorates Estrogen Deficiency-Induced Osteoporosis via Simultaneous Regulation of Osteoblast and Osteoclast Differentiation. ACS Pharmacol Transl Sci 2023;6:270–80. https://doi.org/10.1021/acsptsci.2c00192.

[7] Xue H-Y, Liu M-W, Yang G. Resveratrol suppresses lipopolysaccharide-mediated activation of osteoclast precursor RAW 264.7 cells by increasing miR-181a-5p expression. Int J Immunopathol Pharmacol 2023;37:3946320231154995. https://doi.org/10.1177/03946320231154995.

[8] Wang S, Ma Q, Xie Z, Shen Y, Zheng B, Jiang C, et al. An Antioxidant Sesquiterpene Inhibits Osteoclastogenesis Via Blocking IPMK/TRAF6 and Counteracts OVX-Induced Osteoporosis in Mice. J Bone Miner Res 2021;36:1850–65. https://doi.org/https://doi.org/10.1002/jbmr.4328.

[9] Hong G, Zhou L, Zheng G, Zheng X, Chen Z, He W, et al. A novel Glycyrrhiza glabra extract liquiritin targeting NFATc1 activity and ROS levels to counteract ovariectomy-induced osteoporosis and bone loss in murine model. Front Pharmacol 2023;14:1287827. https://doi.org/10.3389/fphar.2023.1287827.

[10] Li J, Zeng L, Xie J, Yue Z, Deng H, Ma X, et al. Inhibition of Osteoclastogenesis and Bone Resorption in vitro and in vivo by a prenylflavonoid xanthohumol from hops. Sci Rep 2015;5:17605. https://doi.org/10.1038/srep17605.

[11] Tan EM, Li L, Indran IR, Chew N, Yong E-L. TRAF6 Mediates Suppression of Osteoclastogenesis and Prevention of Ovariectomy-Induced Bone Loss by a Novel Prenylflavonoid. J Bone Miner Res Off J Am Soc Bone Miner Res 2017;32:846–60. https://doi.org/10.1002/jbmr.3031.

[12] Ke D, Wang Y, Yu Y, Wang Y, Zheng W, Fu X, et al. Curcumin-activated autophagy plays a negative role in its anti-osteoclastogenic effect. Mol Cell Endocrinol 2020;500:110637. https://doi.org/https://doi.org/10.1016/j.mce.2019.110637.

[13] Kim SC, Kim HJ, Park GE, Pandey RP, Lee J, Sohng JK, et al. Trilobatin ameliorates bone loss via suppression of osteoclast cell differentiation and bone resorptive function in vitro and in vivo. Life Sci 2021;270:119074. https://doi.org/https://doi.org/10.1016/j.lfs.2021.119074.

[14] Kim B, Lee KY, Park B. Icariin abrogates osteoclast formation through the regulation of the RANKL-mediated TRAF6/NF-κB/ERK signaling pathway in Raw264.7 cells. Phytomedicine 2018;51:181–90. https://doi.org/https://doi.org/10.1016/j.phymed.2018.06.020.

[15] Anzai M, Watanabe-Takahashi M, Kawabata H, Mizuno S, Taguchi Y, Inoue J, et al. A tetravalent peptide that binds to the RANK-binding region of TRAF6 via a multivalent interaction efficiently inhibits osteoclast differentiation. Biochem Biophys Res Commun 2022;636:178–83. https://doi.org/https://doi.org/10.1016/j.bbrc.2022.10.075.

[16] Wu M-H, Hsu W-B, Chen M-H, Shi C-S. Inhibition of Neddylation Suppresses Osteoclast Differentiation and Function In Vitro and Alleviates Osteoporosis In Vivo. Biomedicines 2022;10. https://doi.org/10.3390/biomedicines10102355.

[17] Wang N, Hao Y, Fu L. Trimethylamine-N-Oxide Promotes Osteoclast Differentiation and Bone Loss via Activating ROS-Dependent NF-κB Signaling Pathway. Nutrients 2022;14. https://doi.org/10.3390/nu14193955.

[18] Yu J, Hang Y, Sun W, Wang G, Xiong Z, Ai L, et al. Anti-Osteoporotic Effect of Lactobacillus brevis AR281 in an Ovariectomized Mouse Model Mediated by Inhibition of Osteoclast Differentiation. Biology (Basel) 2022;11. https://doi.org/10.3390/biology11030359.

[19] Jang A-R, Park J-S, Kim D-K, Park J-Y, Ahn J-H, Kim D-Y, et al. Cell-free culture supernatant of Lactobacillus curvatus Wikim38 inhibits RANKL-induced osteoclast differentiation and ameliorates bone loss in ovariectomized mice. Lett Appl Microbiol 2021;73:383–91. https://doi.org/10.1111/lam.13525.

[20] Kumar V, Haldar S, Ghosh S, Chauhan S, Sharma A, Dhankhar P, et al. Pterostilbene-isothiocyanate impedes RANK/TRAF6 interaction to inhibit osteoclastogenesis, promoting osteogenesis in vitro and alleviating glucocorticoid induced osteoporosis in rats. Biochem Pharmacol 2022;206:115284. https://doi.org/https://doi.org/10.1016/j.bcp.2022.115284.

[21] Liu J, Ding S, Yang L, Zhao X, Ren R, Wang Y, et al. Integration of pharmacodynamics and metabolomics reveals the therapeutic effects of 6-acetylacteoside on ovariectomy-induced osteoporosis mice. Phytomedicine 2022;106:154399. https://doi.org/https://doi.org/10.1016/j.phymed.2022.154399.

[22] Wang F, Yang G, Li Y, Tang Z, Du J, Song H, et al. A peptide from wheat germ abolishes the senile osteoporosis by regulating OPG/RANKL/RANK/TRAF6 signaling pathway. Phytomedicine 2022;104:154304. https://doi.org/https://doi.org/10.1016/j.phymed.2022.154304.

[23] Wang Q, Wang H, Yan H, Tian H, Wang Y, Yu W, et al. Suppression of osteoclast multinucleation via a posttranscriptional regulation-based spatiotemporally selective delivery system. Sci Adv 2022;8:eabn3333. https://doi.org/10.1126/sciadv.abn3333.

[24] Chen Y, Zhang L, Li Z, Wu Z, Lin X, Li N, et al. Mogrol Attenuates Osteoclast Formation and Bone Resorption by Inhibiting the TRAF6/MAPK/NF-κB Signaling Pathway In vitro and Protects Against Osteoporosis in Postmenopausal Mice. Front Pharmacol 2022;13:803880. https://doi.org/10.3389/fphar.2022.803880.

[25] Zhao M, Mei F, Lu J, Xiang Q, Xia G, Zhang X, et al. Gadus morhua Eggs Sialoglycoprotein Prevent Estrogen Deficiency-Induced High Bone Turnover by Controlling OPG/RANKL/TRAF6 Pathway and Serum Metabolism. Front Nutr 2022;9:871521. https://doi.org/10.3389/fnut.2022.871521.

[26] Qiu Z, Li L, Huang Y, Shi K, Zhang L, Huang C, et al. Puerarin specifically disrupts osteoclast activation via blocking integrin-β3 Pyk2/Src/Cbl signaling pathway. J Orthop Transl 2022;33:55–69. https://doi.org/10.1016/j.jot.2022.01.003.

[27] Lee S, Kim M, Hong S, Kim EJ, Kim J-H, Sohn Y, et al. Effects of Sparganii Rhizoma on Osteoclast Formation and Osteoblast Differentiation and on an OVX-Induced Bone Loss Model. Front Pharmacol 2021;12:797892. https://doi.org/10.3389/fphar.2021.797892.

[28] Wang G, Chen K, Ma C, Wang C, Chen D, He J, et al. Roburic acid attenuates osteoclastogenesis and bone resorption by targeting RANKL-induced intracellular signaling pathways. J Cell Physiol 2022;237:1790–803. https://doi.org/https://doi.org/10.1002/jcp.30642.

[29] Li M, Fu S, Cai Z, Li D, Liu L, Deng D, et al. Dual regulation of osteoclastogenesis and osteogenesis for osteoporosis therapy by iron oxide hydroxyapatite core/shell nanocomposites. Regen Biomater 2021;8:rbab027. https://doi.org/10.1093/rb/rbab027.

[30] Nie C, Hu J, Wang B, Li H, Yang X, Hong F. Effects of Co-exposure to Fluoride and Arsenic on TRAF-6 Signaling and NF-κB Pathway of Bone Metabolism. Biol Trace Elem Res 2023;201:4447–55. https://doi.org/10.1007/s12011-022-03508-9.

[31] Liu L, Jin R, Duan J, Yang L, Cai Z, Zhu W, et al. Bioactive iron oxide nanoparticles suppress osteoclastogenesis and ovariectomy-induced bone loss through regulating the TRAF6-p62-CYLD signaling complex. Acta Biomater 2020;103:281–92. https://doi.org/10.1016/j.actbio.2019.12.022.

[32] Noguchi T, Ebina K, Hirao M, Morimoto T, Koizumi K, Kitaguchi K, et al. Oxygen ultra-fine bubbles water administration prevents bone loss of glucocorticoid-induced osteoporosis in mice by suppressing osteoclast differentiation. Osteoporos Int 2017;28:1063–75. https://doi.org/10.1007/s00198-016-3830-1.

[33] Karim K, Giribabu N, Salleh N. Marantodes pumilum Var Alata (Kacip Fatimah) ameliorates derangement in RANK/RANKL/OPG pathway and reduces inflammation and oxidative stress in the bone of estrogen-deficient female rats with type-2 diabetes. Phytomedicine 2021;91:153677. https://doi.org/10.1016/j.phymed.2021.153677.

[34] Hong J, Shi Z, Li C, Ji X, Li S, Chen Y, et al. Virtual screening identified natural Keap1-Nrf2 PPI inhibitor alleviates inflammatory osteoporosis through Nrf2-mir214-Traf3 axis. Free Radic Biol Med 2021;171:365–78. https://doi.org/10.1016/j.freeradbiomed.2021.05.020.

[35] Sun K, Zhu J, Deng Y, Xu X, Kong F, Sun X, et al. Gamabufotalin Inhibits Osteoclastgenesis and Counteracts Estrogen-Deficient Bone Loss in Mice by Suppressing RANKL-Induced NF-κB and ERK/MAPK Pathways. Front Pharmacol 2021;12:629968. https://doi.org/10.3389/fphar.2021.629968.

[36] Wang K, Chen Y, Gao S, Wang M, Ge M, Yang Q, et al. Norlichexanthone purified from plant endophyte prevents postmenopausal osteoporosis by targeting ER α to inhibit RANKL signaling. Acta Pharm Sin B 2021;11:442–55. https://doi.org/10.1016/j.apsb.2020.09.012.

[37] Xiao L, Zhong M, Huang Y, Zhu J, Tang W, Li D, et al. Puerarin alleviates osteoporosis in the ovariectomy-induced mice by suppressing osteoclastogenesis via inhibition of TRAF6/ROS-dependent MAPK/NF-κB signaling pathways. Aging (Albany NY) 2020;12:21706–29. https://doi.org/10.18632/aging.103976.

[38] Li Y, Li N, Zhao X, Zhang B, Yang L, Liu J, et al. Beneficial effect of 2’-acetylacteoside on ovariectomized mice via modulating the function of bone resorption. Biomed Pharmacother 2020;131:110747. https://doi.org/10.1016/j.biopha.2020.110747.

[39] Ni S, Qian Z, Yuan Y, Li D, Zhong Z, Ghorbani F, et al. Schisandrin A restrains osteoclastogenesis by inhibiting reactive oxygen species and activating Nrf2 signalling. Cell Prolif 2020;53:e12882. https://doi.org/10.1111/cpr.12882.

[40] Kim E-N, Kwon J, Lee H-S, Lee S, Lee D, Jeong G-S. Inhibitory Effect of Cudratrixanthone U on RANKL-Induced Osteoclast Differentiation and Function in Macrophages and BMM Cells. Front Pharmacol 2020;11:1048. https://doi.org/10.3389/fphar.2020.01048.

[41] Zhan Q, Tian Y, Han L, Wang K, Wang J, Xue C. The opposite effects of Antarctic krill oil and arachidonic acid-rich oil on bone resorption in ovariectomized mice. Food Funct 2020;11:7048–60. https://doi.org/10.1039/d0fo00884b.

[42] Qian Z, Zhong Z, Ni S, Li D, Zhang F, Zhou Y, et al. Cytisine attenuates bone loss of ovariectomy mouse by preventing RANKL-induced osteoclastogenesis. J Cell Mol Med 2020;24:10112–27. https://doi.org/10.1111/jcmm.15622.

[43] Jo Y-J, Lee HI, Kim N, Hwang D, Lee J, Lee G-R, et al. Cinchonine inhibits osteoclast differentiation by regulating TAK1 and AKT, and promotes osteogenesis. J Cell Physiol 2021;236:1854–65. https://doi.org/https://doi.org/10.1002/jcp.29968.

[44] Ma W, Wang F, You Y, Wu W, Chi H, Jiao G, et al. Ortho-silicic Acid Inhibits RANKL-Induced Osteoclastogenesis and Reverses Ovariectomy-Induced Bone Loss In Vivo. Biol Trace Elem Res 2021;199:1864–76. https://doi.org/10.1007/s12011-020-02286-6.

[45] Chen H, Fang C, Zhi X, Song S, Gu Y, Chen X, et al. Neobavaisoflavone inhibits osteoclastogenesis through blocking RANKL signalling-mediated TRAF6 and c-Src recruitment and NF-κB, MAPK and Akt pathways. J Cell Mol Med 2020;24:9067–84. https://doi.org/10.1111/jcmm.15543.

[46] Lee SR, Jeon H, Kwon JE, Suh H, Kim B-H, Yun M-K, et al. Anti-osteoporotic effects of Salvia miltiorrhiza Bunge EtOH extract both in ovariectomized and naturally menopausal mouse models. J Ethnopharmacol 2020;258:112874. https://doi.org/10.1016/j.jep.2020.112874.

[47] Chen K, Yan Z, Wang Y, Yang Y, Cai M, Huang C, et al. Shikonin mitigates ovariectomy-induced bone loss and RANKL-induced osteoclastogenesis via TRAF6-mediated signaling pathways. Biomed Pharmacother 2020;126:110067. https://doi.org/10.1016/j.biopha.2020.110067.

[48] Zhi X, Fang C, Gu Y, Chen H, Chen X, Cui J, et al. Guaiacol suppresses osteoclastogenesis by blocking interactions of RANK with TRAF6 and C-Src and inhibiting NF-κB, MAPK and AKT pathways. J Cell Mol Med 2020;24:5122–34. https://doi.org/10.1111/jcmm.15153.

[49] Zhou L, Song H, Zhang Y, Ren Z, Li M, Fu Q. Polyphyllin VII attenuated RANKL-induced osteoclast differentiation via inhibiting of TRAF6/c-Src/PI3K pathway and ROS production. BMC Musculoskelet Disord 2020;21:112. https://doi.org/10.1186/s12891-020-3077-z.

[50] Zhang B, Yang L-L, Ding S-Q, Liu J-J, Dong Y-H, Li Y-T, et al. Anti-Osteoporotic Activity of an Edible Traditional Chinese Medicine Cistanche deserticola on Bone Metabolism of Ovariectomized Rats Through RANKL/RANK/TRAF6-Mediated Signaling Pathways. Front Pharmacol 2019;10:1412. https://doi.org/10.3389/fphar.2019.01412.

[51] Zhi X, Wang L, Chen H, Fang C, Cui J, Hu Y, et al. l-tetrahydropalmatine suppresses osteoclastogenesis in vivo and in vitro via blocking RANK-TRAF6 interactions and inhibiting NF-κB and MAPK pathways. J Cell Mol Med 2020;24:785–98. https://doi.org/10.1111/jcmm.14790.

[52] Ruan L, Jiang N, Guo F, Xu H, Zhang J, Sun J. The antiresoptive effects of recombinant Lingzhi-8 protein against retinoic acid-induced osteopenia. Eur J Pharmacol 2019;863:172669. https://doi.org/https://doi.org/10.1016/j.ejphar.2019.172669.

[53] Sun X, Xie Z, Hu B, Zhang B, Ma Y, Pan X, et al. The Nrf2 activator RTA-408 attenuates osteoclastogenesis by inhibiting STING dependent NF-κb signaling. Redox Biol 2020;28:101309. https://doi.org/https://doi.org/10.1016/j.redox.2019.101309.

[54] Xu W, Zhu X, Chen C, Hu R, Li Y, Xu R, et al. Beneficial effect of pristimerin against the development of osteoporosis in ovariectomy-induced osteoporosis rats by the RANKL/TRAF6/NF-κB pathway. Arch Med Sci 2022;18:1650–8. https://doi.org/10.5114/aoms.2019.86816.

[55] Shang Q, Yu X, Ren H, Shen G, Zhao W, Zhang Z, et al. Effect of Plastrum Testudinis Extracts on the Proliferation and Osteogenic Differentiation of rBMSCs by Regulating p38 MAPK-Related Genes. Evid Based Complement Alternat Med 2019;2019:6815620. https://doi.org/10.1155/2019/6815620.

[56] Chen X, Li X, Zhai X, Zhi X, Cao L, Qin L, et al. Shikimic Acid Inhibits Osteoclastogenesis in Vivo and in Vitro by Blocking RANK/TRAF6 Association and Suppressing NF-κB and MAPK Signaling Pathways. Cell Physiol Biochem Int J Exp Cell Physiol Biochem Pharmacol 2018;51:2858–71. https://doi.org/10.1159/000496039.

[57] Hu B, Sun X, Yang Y, Ying Z, Meng J, Zhou C, et al. Tomatidine suppresses osteoclastogenesis and mitigates estrogen deficiency-induced bone mass loss by modulating TRAF6-mediated signaling. FASEB J 2019;33:2574–86. https://doi.org/https://doi.org/10.1096/fj.201800920R.

[58] Wang H, Feng T, Guo D, Zhang M, Chen L, Zhou Y. Sanggenon C Stimulates Osteoblastic Proliferation and Differentiation, Inhibits Osteoclastic Resorption, and Ameliorates Prednisone-Induced Osteoporosis in Zebrafish Model. Molecules 2018;23. https://doi.org/10.3390/molecules23092343.

[59] Liu X, Gao X, Liu Y, Liang D, Fu T, Song Y, et al. Daphnetin inhibits RANKL-induced osteoclastogenesis in vitro. J Cell Biochem 2019;120:2304–12. https://doi.org/https://doi.org/10.1002/jcb.27555.

[60] Jang S-A, Song HS, Kwon JE, Baek HJ, Koo HJ, Sohn E-H, et al. Protocatechuic Acid Attenuates Trabecular Bone Loss in Ovariectomized Mice. Oxid Med Cell Longev 2018;2018:7280342. https://doi.org/https://doi.org/10.1155/2018/7280342.

[61] Chen X, Zhi X, Yin Z, Li X, Qin L, Qiu Z, et al. 18β-Glycyrrhetinic Acid Inhibits Osteoclastogenesis In Vivo and In Vitro by Blocking RANKL-Mediated RANK-TRAF6 Interactions and NF-κB and MAPK Signaling Pathways. Front Pharmacol 2018;9:647. https://doi.org/10.3389/fphar.2018.00647.

[62] Liu J, Zhang Z, Guo Q, Dong Y, Zhao Q, Ma X. Syringin prevents bone loss in ovariectomized mice via TRAF6 mediated inhibition of NF-κB and stimulation of PI3K/AKT. Phytomedicine 2018;42:43–50. https://doi.org/https://doi.org/10.1016/j.phymed.2018.03.020.

[63] Park B, Song HS, Kwon JE, Cho SM, Jang S-A, Kim MY, et al. Effects of Salvia miltiorrhiza extract with supplemental liquefied calcium on osteoporosis in calcium-deficient ovariectomized mice. BMC Complement Altern Med 2017;17:545. https://doi.org/10.1186/s12906-017-2047-y.

[64] Wu Y-X, Wu T-Y, Xu B-B, Xu X-Y, Chen H-G, Li X-Y, et al. Protocatechuic acid inhibits osteoclast differentiation and stimulates apoptosis in mature osteoclasts. Biomed Pharmacother 2016;82:399–405. https://doi.org/10.1016/j.biopha.2016.05.008.

[65] Tao X, Qi Y, Xu L, Yin L, Han X, Xu Y, et al. Dioscin reduces ovariectomy-induced bone loss by enhancing osteoblastogenesis and inhibiting osteoclastogenesis. Pharmacol Res 2016;108:90–101. https://doi.org/https://doi.org/10.1016/j.phrs.2016.05.003.

[66] Xia G, Zhao Y, Yu Z, Tian Y, Wang Y, Wang S, et al. Phosphorylated Peptides from Antarctic Krill (Euphausia superba) Prevent Estrogen Deficiency Induced Osteoporosis by Inhibiting Bone Resorption in Ovariectomized Rats. J Agric Food Chem 2015;63:9550–7. https://doi.org/10.1021/acs.jafc.5b04263.

[67] Kong X, Wu W, Yang Y, Wan H, Li X, Zhong M, et al. Total saponin from Anemone flaccida Fr. Schmidt abrogates osteoclast differentiation and bone resorption via the inhibition of RANKL-induced NF-κB, JNK and p38 MAPKs activation. J Transl Med 2015;13:91. https://doi.org/10.1186/s12967-015-0440-1.

[68] Yuan F-L, Xu R-S, Jiang D-L, He X-L, Su Q, Jin C, et al. Leonurine hydrochloride inhibits osteoclastogenesis and prevents osteoporosis associated with estrogen deficiency by inhibiting the NF-κB and PI3K/Akt signaling pathways. Bone 2015;75:128–37. https://doi.org/https://doi.org/10.1016/j.bone.2015.02.017.

[69] Xiu Y, Xu H, Zhao C, Li J, Morita Y, Yao Z, et al. Chloroquine reduces osteoclastogenesis in murine osteoporosis by preventing TRAF3 degradation. J Clin Invest 2014;124:297–310. https://doi.org/10.1172/JCI66947.

[70] Li X, He L, Hu Y, Duan H, Li X, Tan S, et al. Sinomenine suppresses osteoclast formation and Mycobacterium tuberculosis H37Ra-induced bone loss by modulating RANKL signaling pathways. PLoS One 2013;8:e74274. https://doi.org/10.1371/journal.pone.0074274.

[71] Kim J-L, Kang M-K, Gong J-H, Park S-H, Han S-Y, Kang Y-H. Novel antiosteoclastogenic activity of phloretin antagonizing RANKL-induced osteoclast differentiation of murine macrophages. Mol Nutr Food Res 2012;56:1223–33. https://doi.org/10.1002/mnfr.201100831.

[72] Zhu L, Wei H, Wu Y, Yang S, Xiao L, Zhang J, et al. Licorice isoliquiritigenin suppresses RANKL-induced osteoclastogenesis in vitro and prevents inflammatory bone loss in vivo. Int J Biochem Cell Biol 2012;44:1139–52. https://doi.org/10.1016/j.biocel.2012.04.003.

[73] Wu X, Li Z, Yang Z, Zheng C, Jing J, Chen Y, et al. Caffeic acid 3,4-dihydroxy-phenethyl ester suppresses receptor activator of NF-κB ligand–induced osteoclastogenesis and prevents ovariectomy-induced bone loss through inhibition of mitogen-activated protein kinase/activator protein 1 and Ca2+–nuclear fac. J Bone Miner Res Off J Am Soc Bone Miner Res 2012;27:1298–308. https://doi.org/10.1002/jbmr.1576.

[74] Kim J-L, Kang S-W, Kang M-K, Gong J-H, Lee E-S, Han SJ, et al. Osteoblastogenesis and osteoprotection enhanced by flavonolignan silibinin in osteoblasts and osteoclasts. J Cell Biochem 2012;113:247–59. https://doi.org/10.1002/jcb.23351.

[75] Kim M, Kim H-S, Kim J-H, Kim E-Y, Lee B, Lee SY, et al. Chaenomelis fructus inhibits osteoclast differentiation by suppressing NFATc1 expression and prevents ovariectomy-induced osteoporosis. BMC Complement Med Ther 2020;20:35. https://doi.org/10.1186/s12906-020-2841-9.

[76] Jeong D-W, Kim E-Y, Kim J-H, Lee B, Hong S, Park JH, et al. Lycopus lucidus Turcz Inhibits the Osteoclastogenesis in RAW 264.7 Cells and Bone Loss in Ovariectomized Rat Model. Evid Based Complement Alternat Med 2019;2019:3231784. https://doi.org/10.1155/2019/3231784.

[77] Han S-Y, Kim Y-K. Yukmijihwang-Tang Suppresses Receptor Activator of Nuclear Factor Kappa-B Ligand (RANKL)-Induced Osteoclast Differentiation and Prevents Ovariectomy (OVX)-Mediated Bone Loss. Molecules 2021;26. https://doi.org/10.3390/molecules26247579.

[78] Kim E-J, Lee H, Kim MH, Yang WM. Inhibition of RANKL-stimulated osteoclast differentiation by Schisandra chinensis through down-regulation of NFATc1 and c-fos expression. BMC Complement Altern Med 2018;18:270. https://doi.org/10.1186/s12906-018-2331-5.
